# Whole genome resequencing of watermelons to identify single nucleotide polymorphisms related to flesh color and lycopene content

**DOI:** 10.1371/journal.pone.0223441

**Published:** 2019-10-09

**Authors:** Saminathan Subburaj, Kayoun Lee, Yongsam Jeon, Luhua Tu, Gilwoo Son, SuBok Choi, Yong-Pyo Lim, Cecilia McGregor, Geung-Joo Lee

**Affiliations:** 1 Department of Horticulture, Chungnam National University, Daejeon, Republic of Korea; 2 Breeding Institute, Hyundai Seed Co Ltd., Yeoju, Gyeonggi, Republic of Korea; 3 Asia Seed, Co., Ltd., Seoul, Republic of Korea; 4 Department of Horticulture, University of Georgia, Athens, GA, United States of America; ICAR-Indian Institute of Agricultural Biotechnology, INDIA

## Abstract

Cultivated watermelon (*Citrullus lanatus*) is one of the most important food crops in the Cucurbitaceae family. Diversification after domestication has led cultivated watermelons to exhibit diverse fruit flesh colors, including red, yellow, and orange. Recently, there has been increased interest in red-fleshed watermelons because they contain the antioxidant *cis*-isomeric lycopene. We performed whole genome resequencing (WGRS) of 24 watermelons with different flesh colors to identify single-nucleotide polymorphisms (SNPs) related to high lycopene content. The resequencing data revealed 203,894–279,412 SNPs from read mapping between inbred lines and the 97103 reference genome. In total, 295,065 filtered SNPs were identified, which had an average polymorphism information content of 0.297. Most of these SNPs were intergenic (90.1%) and possessed a transversion (Tv) rate of 31.64%. Overall, 2,369 SNPs were chosen at 0.5 Mb physical intervals to analyze genetic diversity across the 24 inbred lines. A neighbor-joining dendrogram and principal coordinate analysis (PCA) based on the 2,369 SNPs revealed that the 24 inbred lines could be grouped into high and low lycopene-type watermelons. In addition, we analyzed SNPs that could discriminate high lycopene content, red-fleshed watermelon from low lycopene, yellow or orange watermelon inbred lines. For validation, 19 SNPs (designated as WMHL1–19) were chosen randomly, and cleavage amplified polymorphic sequence (CAPS) markers were designed. Genotyping of the above 24 lines and 12 additional commercial cultivars using WMHL1–19 CAPS markers resulted in match rates of over 0.92 for most validated markers in correlation with the flesh color phenotypes. Our results provide valuable genomic information regarding the high lycopene content phenotype of red-fleshed cultivated watermelons, and the identified SNPs will be useful for the development of molecular markers in the marker-assisted breeding of watermelons with high lycopene content.

## Introduction

Watermelon (*Citrullus lanatus*) is one of the most important horticultural crops in the Cucurbitaceae family. Watermelon originated from north Africa more than 4000 years ago and is now grown throughout the world for its fleshy and sweet fruit [1, 2]. Currently, watermelon-breeding programs are focused on improving diverse traits of interest, which are required for the global seed market. These include the fruit size, skin color, flesh color, maturity, sugar content, resistance to biotic or abiotic stresses, and consumer demand for health-enhancing functions. A major challenge for breeders is the ability to dissect genetic relationship between the cultivated watermelons prior to initiating hybridization as they exhibit narrow genetic diversity [3, 4], resulting in progenies exhibiting unwanted agronomic characteristics derived from crosses of similar backgrounds. Therefore, understanding the population structure of cultivated and parental germplasm is important for modern watermelon breeding. Some progress has been made with the advent of modern molecular tools and their recent developments, which have allowed researchers to dissect the genetic basis of trait inheritance in watermelon [5]; however, many problems are yet to be solved completely.

During the 1980s [6], marker-assisted selection (MAS) was proposed for plant breeding and later considered to have great reliability and repeatability [7]. Unlike other marker systems, single nucleotide polymorphisms (SNPs) and cleaved amplified polymorphic sequence (CAPS) offer many advantages, which have enabled the large-scale genotyping and fine mapping of important traits in cultivated watermelons [8, 9]. The recent advent of next-generation sequencing (NGS) technology and high-throughput genotyping methods, which obtain DNA sequence information from the genome of cultivated watermelons, resulted in the production of a novel set of single nucleotide polymorphism (SNP) markers. Following the recent publication of the complete watermelon (97103 v1) genome sequence (http://www.icugi.org/cgi-bin/ICuGI/genome), it is now possible to identify potential molecular markers from the informed selection of genes.

Guo et al. [10] developed 6,784,860 candidate SNP markers through the whole genome resequencing (WGRS) of 20 inbred watermelon lines. Subsequently, Nimmakayala et al. [11] and Reddy et al. [12] identified 11,485 and 13,693 SNPs, respectively, using the genotyping by sequencing (GBS) approach in 183 and 86 watermelon accessions. In addition to the availability of cost-effective DNA sequencing and the complete genome sequence, a novel SNP-based marker platform, DArTseq, has also been used to identify SNP markers across various watermelon accessions to analyze genetic diversity [13, 14]. These SNP markers have subsequently been mapped onto chromosomes of watermelon, and facilitated the construction of high-density genetic linkage maps, which could be useful for identification of quantitative trait loci (QTL), target genes for trait identification, and MAS [11–13].

The carotenoid biosynthesis pathway and its involvement in fruit color has been well characterized in tomato and citrus [15, 16]. Carotenoids can be classified into oxygenated (lutein, violaxanthin, and neoxanthin) and non-oxygenated (β-carotene and lycopene) carotenoids. The formation of lycopene is a major step in carotenoid biosynthesis, in which lycopene β-cyclase (LCY-B) and lycopene ε-cyclase (LYC-E) enzymes are involved in the formation of lycopene, and LYC-B is involved in the formation of β-carotene. In watermelon fruit, flesh color is an important trait, which can be divided into red, yellow (salmon and canary), and orange [17]. Flesh color variations are caused by the accumulation of carotenoid pigments such as lycopene (red), xanthophylls and β-carotene (yellow), and prolycopene and β-carotene (orange) in chromoplast cells of the watermelon flesh [18, 19]. In addition, these carotenoids provide beneficial effects on the human diet; in particular, lycopene, which constitutes 84–97% of the total carotenoids in red color watermelon fruits [18, 20], has been reported to be involved in the prevention of cancers and cardiovascular diseases [21, 22]. This has reinforced the development of novel fruit-related traits through conventional and modern molecular breeding strategies in watermelon [12, 23–25].

Poole [26] reported that the inheritance of watermelon flesh colors, such as red and canary yellow, are controlled by loci *c* and *C* (dominant to *c*), respectively. Henderson [27] reported that red, yellow, and orange flesh colors are regulated by three color determinant loci, namely *Y* (dominant to *y* and *y*^o^), *y* (yellow), and *y*^o^ (dominant to *y*), respectively. Furthermore, an interaction between the *C* and *I* loci was found to result in red flesh, where the *C* locus is either allele *C* or *c*, which is inhibited when the *I* locus is in the homozygous recessive allelic (*i*) form [28]. Although evidence indicates that these loci are involved in flesh color, the regulatory mechanisms underlying flesh color formation in watermelon remain largely unknown. Bang et al. [19, 29, 30] investigated flesh inheritance in watermelon through the characterization of a SNP mutation on the *lycopene beta-cyclase* (*LCYB*) gene, and its expression between red and canary yellow watermelon, and developed the Clcyb.600 marker, which perfectly co-segregated with flesh color phenotypes. Recently, Zhu et al. [31] ruled out comparative transcriptional regulation between red and pale-yellow watermelons during fruit development and ripening stages and identified a large number of upregulated genes related to carotenoid biosynthesis and plant hormone pathways.

Several QTLs associated with flesh color have been detected by various researchers. Two QTLs have been identified for red flesh color on chromosomes 2 and 8 [32]. Recently, QTLs for lycopene content (LCYB) in watermelon was identified on chromosome 4 [8, 33] and for β-carotene on chromosome 1 [25]. Although several genes and QTLs responsible for flesh color variations have been identified, applicable markers in watermelon breeding for red flesh color or high lycopene content remain limited and further polymorphic markers need to be saturated to enhance the linkage mapping for those traits. With the availability of the complete watermelon genome sequence [10], it has become feasible to develop a wide range of molecular markers to facilitate the selection of desired traits. The objectives of our study were (1) to perform WGRS on 24 inbred watermelon lines with different fruit traits to identify genome-wide SNP markers, (2) examine the use of identified genome-wide SNPs to elucidate genetic relationships between the inbred lines, and (3) rapidly develop diagnostic CAPS markers closely linked to red flesh color-type cultivars for large-scale MAS in watermelon.

## Materials and methods

### Plant materials and genomic DNA extraction

In total, 24 watermelon inbred lines were obtained from two domestic seed companies in Korea (Hyundai and Asia Seed Company) for WGRS analysis. The collected inbred lines possessed diverse fruit characteristics, including shape (circular and elliptic), fruit ground color (dark green, green, light green, and yellow), stripes (inconspicuous or solid [no stripe], week, medium, and strong [jubilee or crimson type]), and flesh color (red, yellow, and orange), as indicated in Table 1 and S1A Fig. according to UPOV [34]. To validate the developed markers, an additional 12 commercial watermelon cultivars with red (4), yellow (4), and orange (4) flesh colors were used (S1 Table and S1B Fig). Seedlings of inbred lines were grown in 72-cell polyethylene flats under greenhouse conditions. After the appearance of the second and third true leaves, leaf samples were collected for DNA extraction using the cetyl trimethyl ammonium bromide (CTAB) method [35]. The quality and quantity of purified genomic DNA were further assessed by spectrophotometry (Infinium F-200; Nanodrop, Illumina Inc, San Diego, CA, USA) and fluorometry (Qubit; Thermo Fisher Scientific Inc, Waltham, MA, USA), followed by 0.7% agarose gel electrophoresis.

**Table 1:** List of 24 watermelon lines used in this study for genome resequencing and their representative fruit characteristics.

Serial no.	Line (generation)	Source	Fruit shape	Ground skin color	Stripe	Flesh color	Growth type
1	801 (F8)	Hyundai Seeds	Circular	Dark green	Solid	Red	Vine
2	802 (F8)	Hyundai Seeds	Circular	Dark green	Solid	Red	Vine
3	803 (F8)	Hyundai Seeds	Circular	Green	Jubilee	Red	Vine
4	812 (F8)	Hyundai Seeds	Circular	Green	Jubilee	Red	Vine
5	829 (F8)	Hyundai Seeds	Elliptic	Green	Jubilee	Red	Vine
6	830 (F8)	Hyundai Seeds	Elliptic	Dark green	Crimson	Red	Vine
7	832 (F8)	Hyundai Seeds	Elliptic	Green	Solid	Red	Vine
8	917 (F4)	Hyundai Seeds	Circular	Green	Jubilee	Red	Vine
9	45 (F6)	Asia Seeds	Circular	Green	Jubilee	Red	Bush
10	3 (F8)	Asia Seeds	Circular	Green	Jubilee	Yellow	Bush
11	816 (F8)	Hyundai Seeds	Circular	Green	Jubilee	Yellow	Vine
12	819 (F8)	Hyundai Seeds	Circular	Yellow	Solid	Yellow	Vine
13	833 (F8)	Hyundai Seeds	Elliptic	Green	Jubilee	Yellow	Vine
14	834 (F8)	Hyundai Seeds	Elliptic	Green	Jubilee	Yellow	Vine
15	835 (F5)	Hyundai Seeds	Elliptic	Dark green	Solid	Yellow	Vine
16	837 (F8)	Hyundai Seeds	Elliptic	Dark green	Solid	Yellow	Vine
17	838 (F8)	Hyundai Seeds	Elliptic	Dark green	Solid	Yellow	Vine
18	919 (F4)	Hyundai Seeds	Circular	Yellow	Jubilee	Yellow	Vine
19	1 (F8)	Asia Seeds	Circular	Green	Jubilee	Orange	Bush
20	29 (F8)	Asia Seeds	Elliptic	Light green	Solid	Orange	Bush
21	820 (F8)	Hyundai Seeds	Circular	yellow	Jubilee	Orange	Vine
22	840 (F8)	Hyundai Seeds	Elliptic	Light green	Solid	Orange	Vine
23	842 (F8)	Hyundai Seeds	Elliptic	Dark green	Solid	Orange	Vine
24	843 (F8)	Hyundai Seeds	Elliptic	Dark green	Solid	Orange	Vine

### Estimation of fruit carotenoid and color coordinates

As listed in Table 1, within each flesh color group (red, yellow and orange), correlations between inbred lines were observed for traits that likely share common fruit characteristics, such as shape, skin color, stripe pattern, and flesh color. Thus, we randomly selected 13 inbred lines (four or five inbred lines per flesh group) with priority given to the flesh color rather than the other characteristics (Table 1). For 13 of these inbred lines (Red: 802, 803, 829, 830, and 917; Yellow: 834, 835, 838, and 919; and Orange: 820, 840, 842, and 843), flesh color and major fruit carotenoids (including lycopene, β-carotene, and phytoene) were measured 40–45 days post-anthesis (DPA). One fruit from each line was harvested and transported to the laboratory to prevent internal bruising. Fruits were sliced and sampled immediately to avoid carotenoid degradation. The color coordinates were measured at five different regions [20], including the center and four other locular regions from the center of the fruit in cross-section, using an 8-mm aperture of the chromatic color difference colorimeter of CR—400/410 (Konica Minolta, Japan). Measurements were performed in the CIELAB color space, and the spectrum of color space values were recorded, including L* (light/dark), a* (green/red), b* (yellow/blue), and ΔE*ab (total color differences). Then, flesh samples of the same fruits were used to quantify the carotenoids. Approximately 40–50 g of flesh samples were collected (diameter of 2 cm) from each region and homogenized to a fine puree. Puree from each region from those lines was combined into one composite, for which the average carotenoid profiles were determined from two random samples of homogenized puree according to the methods previously described [20].

### Statistical analysis

Statistical analysis for color parameters were conducted using an SPSS 19.0 statistical software package. Significance was analyzed using one-way analysis of variance (ANOVA) followed by Tukey's multiple comparison test at the P < 0.05 level. The carotenoid contents among lines in the red, yellow, and orange flesh color groups were compared by calculating the standard error of the mean.

### Whole genome resequencing on watermelon inbred lines and SNP calling

WGRS of inbred watermelon lines was performed through NGS using the Hiseq 2000 and Nextseq (Illumina, San Diego, CA, USA) systems at Seeders company (http://tgsol.seeders.co.kr/, Korea). The generated Illumina paired-end sequence reads for each inbred line were represented approximately 20× genome coverage considering the reference watermelon (97103) genome size (350 Mb), as previously described [24]. In order to filter out the duplicated read-pairs in Illumina reads, Mark Duplicates in Picard tools (V1.106) was deployed. Then, the non-redundant reads were pre-processed with a quality check, followed by trimming. The SolexaQA software package (v.1.13) was used for read trimming with the following criteria: (1) 0.05 probability of error, (2) Phred quality score Q ≥ 20 (dynamic trim either end of the reads when the score dropped below Q = 20), and (3) minimum length of short read ≥ 25 bp. After low-quality reads were removed during trimming, reads were subjected to further analysis.

To identify raw SNPs, the reads were mapped onto the recently released *C*. *lanatus* [10] watermelon (97103) reference genome (v1) using Burrows–Wheeler alignment (BWA) software [36], resulting in the creation of SAM files of aligned reads. SAM files were sorted (using Picard tools v1.118) and then converted to BAM files (using SAMtools v0.1.19) [37] for SNP variant calling (with default parameters in the mpileup application in SAMtools). To prepare an SNP matrix of 24 watermelon lines, the SNP variants were further filtered based on the following criteria: (1) unmapped reads, (2) read depth < 3, (3) mapping quality < 30, and (4) biallelic SNP (InDel). The filtered SNP variants were extracted into a Variant Call File (VCF) format and classified as homozygous (≥ 90% of reads), heterozygous (40% to 60% of reads), and others (indistinguishable as either homozygous or heterozygous).

### Selection of SNPs for marker-assisted breeding, genetic relationships, and genome-wide SNP distribution analysis in 24 inbred lines

The identified SNP variants were further annotated as genic (within genes) or intergenic (other genomic) SNP variants. Genic SNP variants were further categorized into coding sequences of exon and intron. The polymorphism information content (PIC), an index of polymorphism, was calculated for SNP variants from the 24 watermelon lines [38]. The above details were used for marker development with the following criteria: (1) among SNP variants evenly distributed on chromosomes (0 to 11) at a physical interval of 0.5 Mb, (2) with a read depth > 3, (3) with a PIC value > 0, and (4) located in genic regions. In this way, we produced a subset of SNPs, which was later used for the evaluation of genetic relationships and SNP distribution in 24 inbred lines.

To elucidate the genetic relationship between 24 inbred watermelon lines, a principal component analysis (PCA) was performed based on a subset of the total SNPs using the package SNPRelate in R [39]. Pairwise genetic distances for the 24 resequenced inbred lines were estimated based on the total SNP subset using identity by state (IBS) similarity in the TASSEL 5.0 software [40]. A tree was built using the neighbor-joining (NJ) algorithm with Pearson’s product-moment correlation coefficient (*r*) in MEGA 7.0.14 software [41]. SNPs, insertions, and deletions (InDel) within a 100 kb interval were counted for filtered total SNPs and the total SNP subset, and then plotted on chromosomes using Circos software (http://www.circos.ca/). The physical location of major QTLs for various fruit trait-related characteristics, including flesh color and lycopene content, were obtained from previous reports [8, 25, 42, 43] and visualized on the Circos plot.

### Development of CAPS markers specific to high lycopene content, primer design, and validation of DNA polymorphisms

Based on the preliminary visual assessment and a cross-validation by estimation of color coordinates, the 24 inbred lines were classified according to the color of their fruit flesh (Table 1). Among different flesh-colored watermelons, red-fleshed watermelons contain high levels of lycopene, which is the most abundant fruit carotenoid [44]. Therefore, we searched for the red flesh-specific SNP in the SNP subset matrix by identifying protein-coding genes bearing SNPs that were 1) monomorphic among red flesh type, 2) monomorphic among other (n = 15) non-red types, such as yellow and orange, and 3) polymorphic between red-type and non-red-type inbred lines. To develop CAPS markers from high-throughput sequencing data, the sequences of selected SNP variants were analyzed in SNP2CAPS software [45] with an input parameter of 22 different restriction enzymes (*AccI*, *AluI*, *ApeKI*, *AvaI*, *BamHI*, *BbsI*, *BsrI*, *Bsu36I*, *DpnI*, *EcoNI*, *EcoRI*, *Hind-III*, *MluI*, *MspI*, *NdeI*, *NruI*, *PstI*, *SacI*, *SalI*, *SpeI*, *XbaI*, and *XhoI*). Following prediction of the enzyme site, primers were designed for candidate CAPS sequences using Primer3Plus software with the following criteria: oligo length of 18–24 bp, GC content of 40–60%, Tm of 55–60°C, and amplicon length of 100–500 bp.

For experimental validation of CAPS markers, each PCR was carried out in a 12 μL reaction volume containing 100 ng of template DNA, 1 μL of 100 μM primers, 1.2 μL of 10× PCR buffer, 1 μL of 10 mM dNTP, and 0.08 μL 5 U/μL Tag, and ddH_2_O to a 12 μL volume. Amplifications were conducted with an initial denaturation step of 95°C for 3 min, which was followed by 40 cycles of 30 s at 95°C, 30 s at 58°C, 30 s at 72°C; and a final cycle of 10 min at 72°C. For the enzyme digestion of PCR amplicons, a 15 μL reaction mixture was used containing: 12 μL of PCR product, 1.5 μL of 10× enzyme buffer, 1 U of restriction enzyme, and ddH_2_0 to a final volume of 15 μL, which was then incubated at 37°C for 2–4 h. Digested products were analyzed by electrophoresis in a 3% metaphor agarose gel (Lonza, Rockland, ME USA.) containing 1× Tris-Borate-EDTA (TBE) buffer, followed by fragment separation at 180 V for 25 min. After the digestion of PCR products and subsequent separation of fragments in agarose gel, the match rate between SNP information and CAPS genotyping was analyzed, as described in a previous study [24].

## Results and discussion

### Assessment of flesh color phenotypes

Cross hybridization between different inbred lines results in genetic populations with different carotenoid profiles, and consequently, the flesh color variations often seen in the pericarp tissues of watermelon fruit [20]. To investigate the biochemical basis, and to confirm variations in flesh color in our inbred watermelon lines, we determined the color space values and major carotenoids, including lycopene, β-carotene, and phytoene at 45 DPA of fruit development across 14 typical inbred lines with different flesh colors.

Statistically significant (P < 0.05) differences were observed for all color parameters (Table 2). All the tested inbred lines showed distinct color parameters, except for the yellow flesh type of 834, which exhibited nonsignificant values that highly differed from the color space values in other yellow flesh types. In the color component of luminosity, such as the light/dark spectrum (L*-values), red flesh types presented a maximum value of 48.47, which was significantly lower than those of both yellow (68.11) and orange (67.42) (P < 0.05). Red/green (a*) values for the red flesh types ranged between 17.66 and 20.94, where yellow and orange flesh types presented maximum values of 3.45 and 5.86, respectively. Conversely, yellow (26.83) and orange (23.56) flesh types possessed a highest value when compared with red flesh (9.56) for yellow/blue (b*) values. Furthermore, analysis of the total color change parameter (ΔE*ab) depicted a highly significant difference (P < 0.05) between red (21.95–24.47) and yellow (7.72–26.26) or orange (3.13–7.59) flesh types as noted for a* or b* color components (Table 2). During the ripening stages of tomato fruit, both the meso and exocarp fruit tissues exhibited both decreased L* and b* values, and increased a* values in the fully ripened red color fruits [46]. This suggests that the observed color space characteristics of inbred watermelon lines with red flesh color are consistent with those in reported studies.

**Table 2:** Estimation of color coordinates and mean color space (ΔE*ab) values in the fruit flesh of 12 watermelon lines.

Inbred line	L*	a*	b*	ΔE*ab	Flesh color
803	48.08 ± 0.77^c^	20.94 ± 1.16^a^	8.96 ± 0.31^c^	24.47 ± 0.55^a^	Red
829	46.22 ± 1.02^c^	18.34 ± 1.31^a^	9.30 ± 0.79^c^	24.01 ± 1.18^a^	Red
830	48.47 ± 0.54^c^	19.34 ± 1.18^a^	9.56 ± 0.26^c^	21.95 ± 0.61^a^	Red
917	45.12 ± 1.05^c^	17.66 ± 1.21^a^	9.17 ± 0.46^c^	23.89 ± 1.63^a^	Red
834	45.76 ± 1.05^c^	3.45 ± 0.30^bcd^	3.96 ± 1.01^c^	26.26 ± 1.46^a^	Yellow
835	63.57 ± 1.74^ab^	1.56 ± 0.60^cde^	26.83 ± 2.36^a^	7.72 ± 0.86^bc^	Yellow
838	64.45 ± 1.68^ab^	–0.66 ± 0.25^e^	17.83 ± 0.49 ^b^	11.33 ± 0.58^b^	Yellow
919	68.11 ± 1.15^a^	0.60 ± 0.22^de^	24.13 ± 2.18^ab^	9.89 ± 1.28^b^	Yellow
820	60.64 ± 2.83^b^	4.37 ± 0.57^bc^	19.09 ± 2.53^b^	7.59 ± 2.62^bc^	Orange
840	67.42 ± 2.08^ab^	2.52 ± 0.15^bcde^	20.99 ± 1.74^ab^	7.73 ± 1.26^bc^	Orange
842	61.01 ± 1.55^ab^	5.86 ± 0.29^b^	18.73 ± 1.56^b^	5.97 ± 1.65^bc^	Orange
843	62.83 ± 1.49^ab^	5.50 ± 0.09^b^	23.56 ± 0.86^ab^	3.13 ± 0.73^c^	Orange

Note:

^a-e^ Different superscripts within columns indicate statistically significant differences (P < 0.05) with one-way ANOVA and Tukey's post-hoc test.

**Fig 1:**
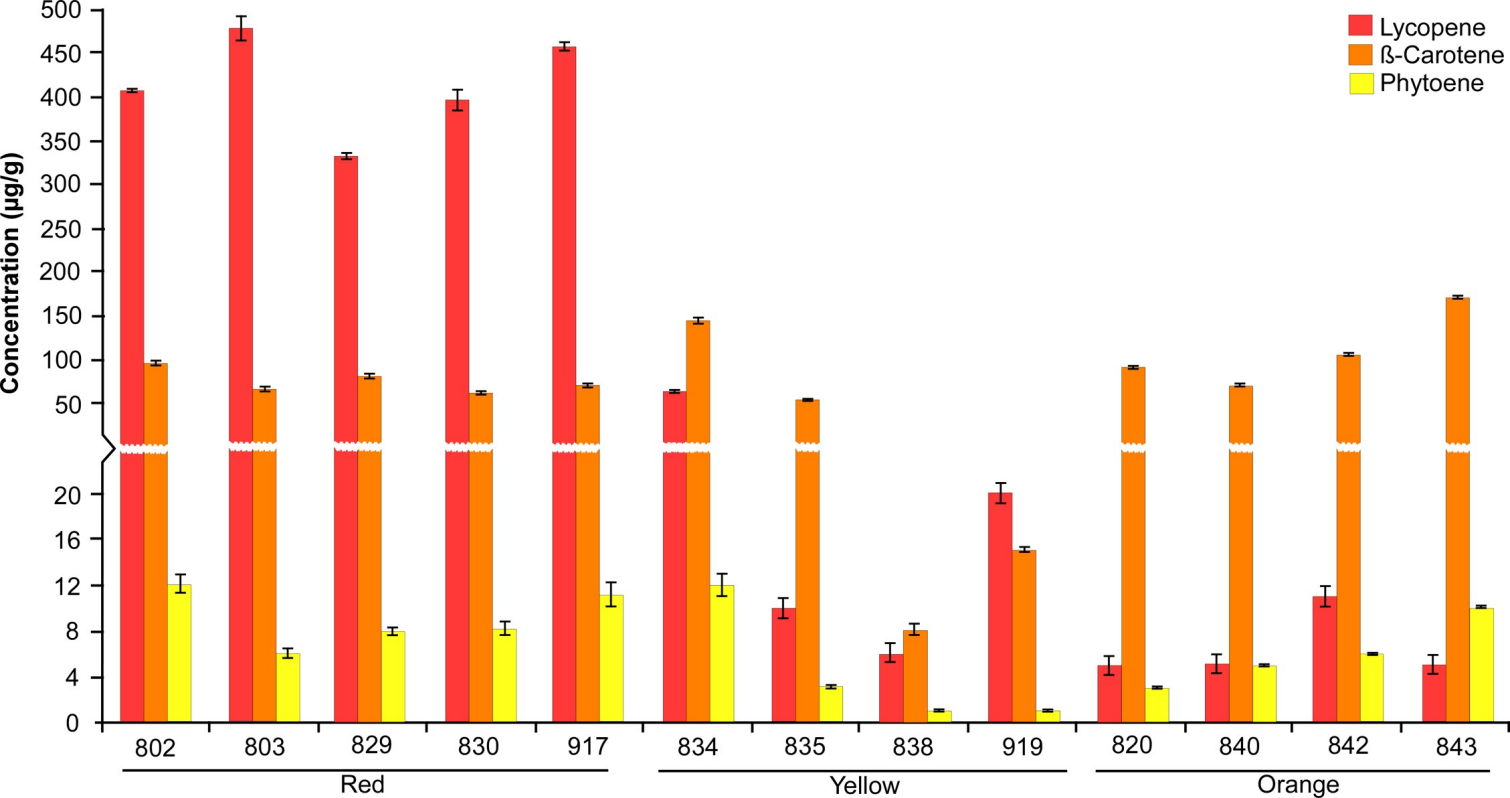
Estimation of major fruit carotenoids. The concentrations of the major carotenoids lycopene, β-carotene, and phytoene (μg g^–1^ ± SE) in 14 watermelon lines as determined at 45 days post-anthesis (DPA).

The major carotenoids, comprising lycopene, β-carotene, and phytoene, are responsible for the red, orange, and yellow flesh colors of watermelon, respectively [19]. With respect to the carotenoid composition of inbred lines, an elevated level of lycopene was noted in all inbred red flesh lines, with maximum and minimum levels of 477 μg/g (line 803) and 333 μg/g (line 829), respectively (Fig 1). This corresponds well with the results of earlier studies, in which the major carotenoid in red watermelon fruits was lycopene [19, 20]. Regarding phytoene, only trace amounts were found in all 13 lines, with the highest level of 12 μg/g observed in line 834 (yellow). An orange-fleshed watermelon (NY162003) has previously been reported to contain mainly β-carotene (>99%), with traces of lycopene and phytoene [18]. These observed carotenoid patterns were confirmed in the present study, where the β-carotene levels were considerably (91–171 μg/g) higher than those of lycopene (5–11 μg/g) and phytoene (3–10 μg/g) in all four orange inbred lines. Therefore, as shown in the Fig 1, the broad range of carotenoid contents observed among the different flesh types suggested that the cutoff values for useful levels of high and low lycopene content could be > 333 μg/g and < 65 μg/g, respectively. Taken together, the color space (ΔE*_ab_) values and carotenoid contents revealed substantial variation among red, yellow, and orange inbred lines (Table 2 and Fig 1). This confirms that the selected inbred watermelon lines possessed different fruit flesh color phenotypes and are, therefore, suitable for further study.

### Whole genome resequencing in 24 watermelon lines

NGS-based WGRS strategies have been widely applied for genetic studies of important traits in many crops [47]. To understand the genetic basis of flesh color variation in watermelon, we performed an NGS-based WGRS to analyze 24 watermelon lines (Table 1). WGRS of genomic DNA from 24 inbred lines produced raw reads ranging from 34.8 × 10^6^ (line 834) to 41.9 × 10^6^ (line 842), with an average length of 101 bp per inbred line. The total length of reads ranged between 3,518,799,499 bp (line 834) and 4,236,980,805 bp (line 842) (S2 Table).

After the trimming of raw reads, the ratio of the total length of raw reads over the trimmed reads (e.g., trimmed/raw) was calculated, and varied from 59.8% (54) to 66.46% (843) (S3 Table), whereby a total genome coverage range of 10.18–12.62× was noted for all inbred lines. Later, these trimmed reads were taken for subsequent analyses. All resequenced data from this study were deposited at GenBank under the accession number PRJNA516776 (http://www.ncbi.nlm. nih.gov/bioproject/ PRJNA516776).

### Identification and characterization of SNP markers

**Fig 2:**
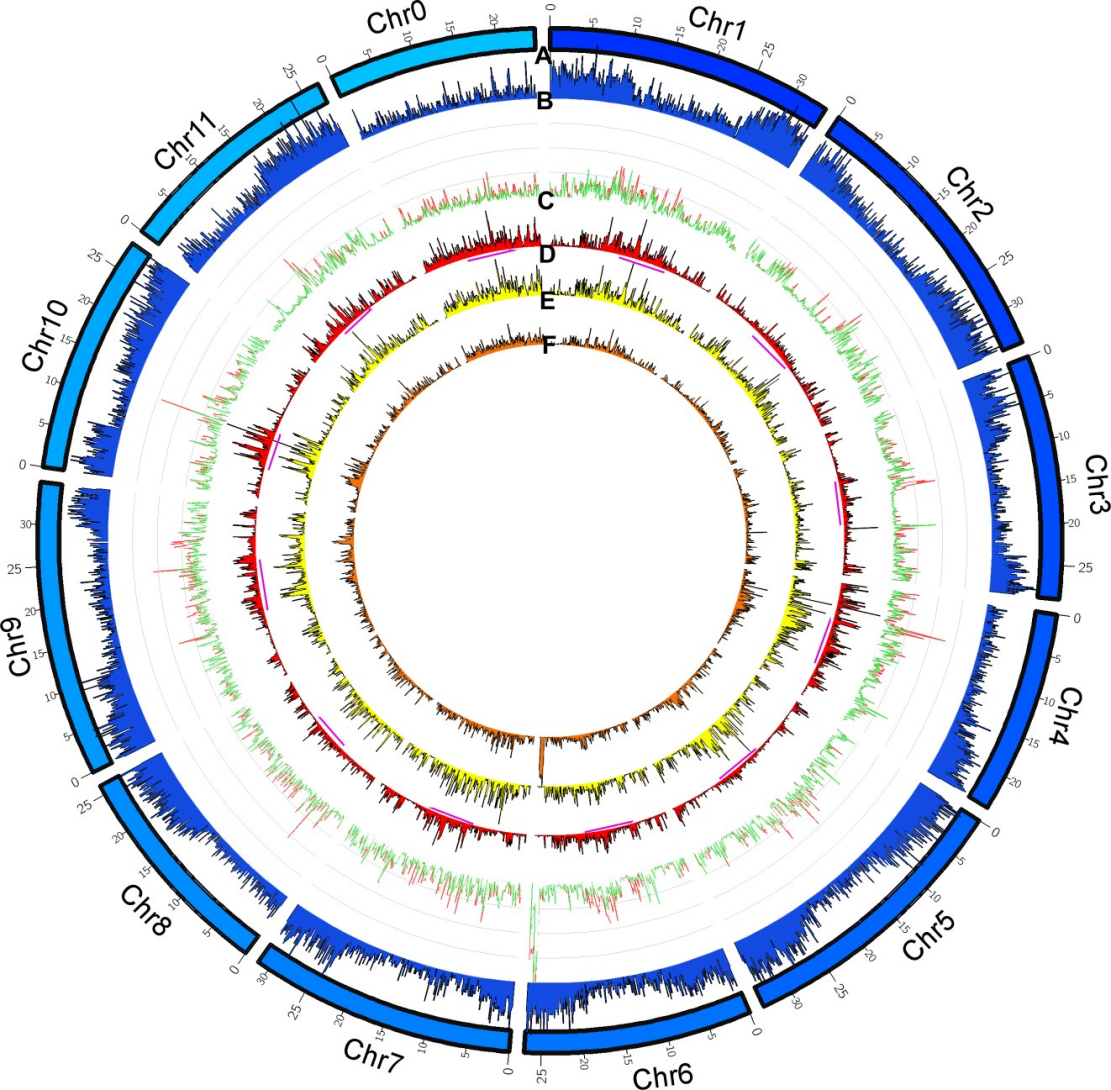
Circos plot showing the chromosomal mapping of identified SNPs/insertion/deletion polymorphisms (InDels) on reference watermelon (97103) chromosomes. (A) chromosome numbers, (B) distribution of coding sequences, (C) line plots of filtered SNPs (colored in *green*) and InDels (colored in *red*) during WGRS of 24 lines with respect to the reference genome 97103, (D–F) histograms of identified SNPs for the 803 (red), 835 (yellow), and 840 (orange) inbred lines. The *magenta* lines beneath the histogram D indicates the centromeric region on each chromosome with higher frequencies of SNPs, which can also be applied to other histograms (Circle E–F).

SNP markers are co-dominant, highly informative, and polymorphic. These attributes make SNPs effective DNA-based molecular markers for studying genome association and genetic diversity in crop breeding [48]. After the removal of non-specific reads, high-quality filtered reads were mapped to the 97103 reference genome. Mapping of reads revealed that resequenced inbred lines consisted of SNPs ranging from 203,894 to 279,412. Following SNP variant calling and subsequent filtering of false positive variants (>5% of minor allele frequency), 295,065 filtered SNPs were identified by comparing the resequenced genome of 24 inbred lines with the reference genome of watermelon (97103). The watermelon has 11 chromosomes, and the distribution of identified SNPs varied greatly as a reflection of chromosome size [10]. The highest number of SNPs (34,488) were found on chromosome 9 (~34 Mb), and the lowest number (15,360) found on chromosomes 11 (~27 Mb) (Fig 2).

The rates of transitions (Ts) and transversions (Tv) were 68.36% and 31.64%, respectively. The SNP PIC values ranged from 0.1 to 0.38, with an average value of 0.297. Most SNPs were in intergenic regions (90.1%), with the remaining (9.9%) located within genic regions. Most (70.7%) SNPs within genic regions were in introns rather than exons. To determine the possible linked functions of SNPs located within genic regions, we searched the corresponding transcripts of genic SNPs. Accordingly, we identified 7712 non-redundant transcripts, and Gene Ontology (GO) annotations were simultaneously performed. Based on GO analysis, these transcripts were classified into “cellular components (79%)”, “molecular functions (73%)”, and “biological processes (72%)”, and were annotated to “primarily located in intracellular components, nucleus, and cytoplasmic part”; “proteins involved in enzyme binding, protein binding, and DNA binding”; and “involved in cellular and metabolic processes and protein metabolism”, respectively (S2 Fig).

### Mining of genome-wide SNP markers and genetic diversity between the resequenced lines

**Fig 3:**
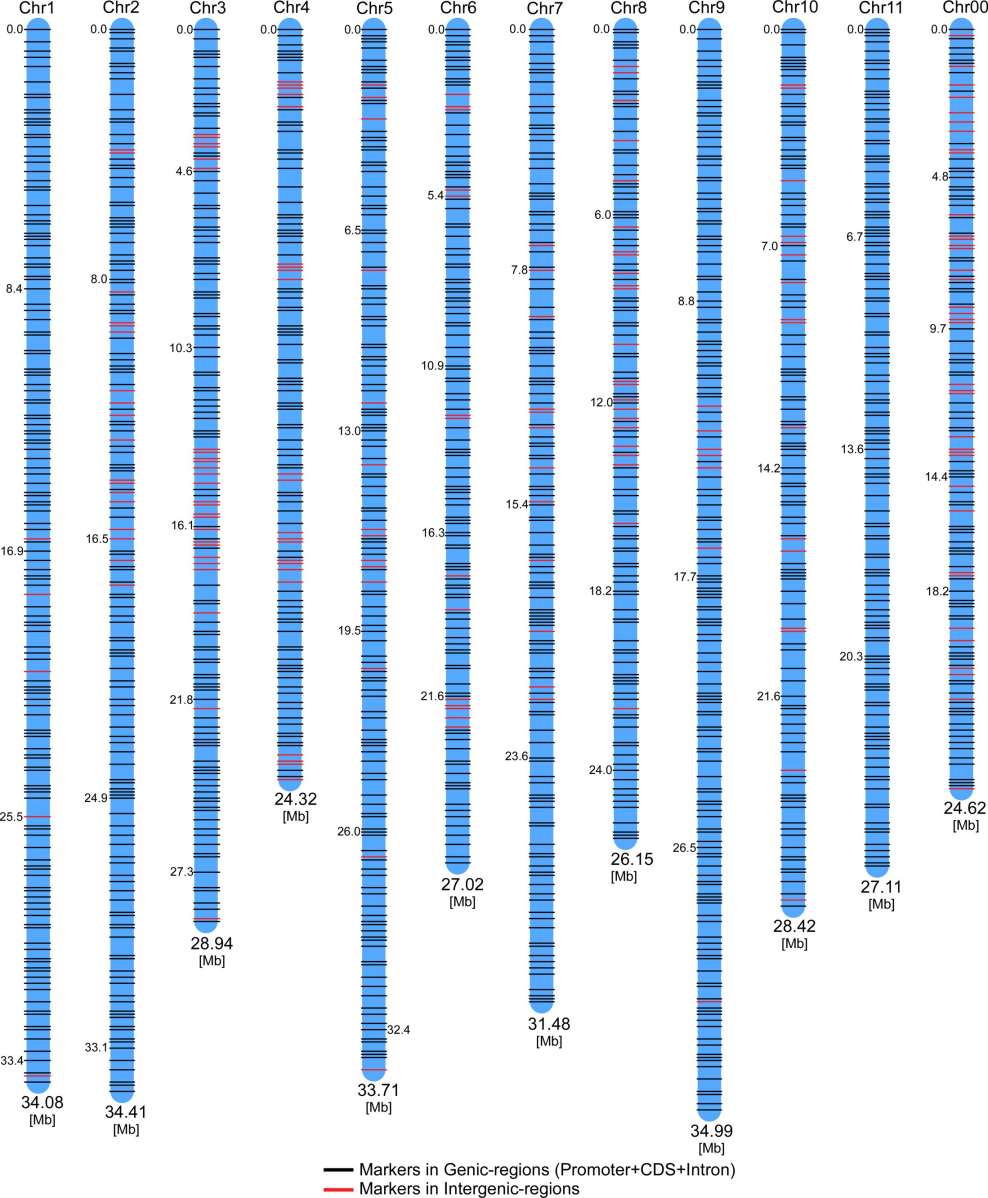
Genomic distribution of selected SNP markers. A total of 2369 SNPs that were identified by the WGRS of 24 lines and selected for the development of molecular markers related to high lycopene content in watermelon.

PIC indicates the usefulness of a marker for detecting polymorphism. Multi-allelic molecular markers, such as RAPD, and AFLP, usually have PIC values that range between 0.5 and 1.0. In contrast, SNPs are bi-allelic in nature, possess a co-dominant inheritance pattern, and have lower PIC values, which can range between 0 and 0.5 [24]. SNPs with low PIC values have been found to efficiently discriminate watermelon genotypes [14, 24]. Therefore, in the present study, we selected 2369 SNPs with lower PIC values (0.1–0.38) to discriminate the genotypes of our 24 inbred lines. Of the 2369 selected SNPs, 2075 were within genic regions (exon and intron) and 294 were in intergenic regions (Table 3 and S4 Table); all the SNPs were evenly distributed on watermelon chromosomes 0–11 at physical intervals of ~0.5 Mb (Fig 3). Details of the selected SNPs and their locations are listed in S4 Table.

**Table 3:** Selected SNP markers for the specific identification of watermelon genotypes with high lycopene contents.

Chr. #	No. of selected SNPs	No. of selected SNPs in intergenic region	No. of selected SNPs in genic region (Promoter + CDS + Intron)
Chr1	228	7	221
Chr2	229	29	200
Chr3	193	32	161
Chr4	163	25	138
Chr5	221	19	202
Chr6	180	17	163
Chr7	210	17	193
Chr8	176	29	147
Chr9	233	9	224
Chr10	188	23	165
Chr11	182	41	141
Chr00	166	46	120
**Total**	**2369**	**294**	**2075**

**Fig 4:**
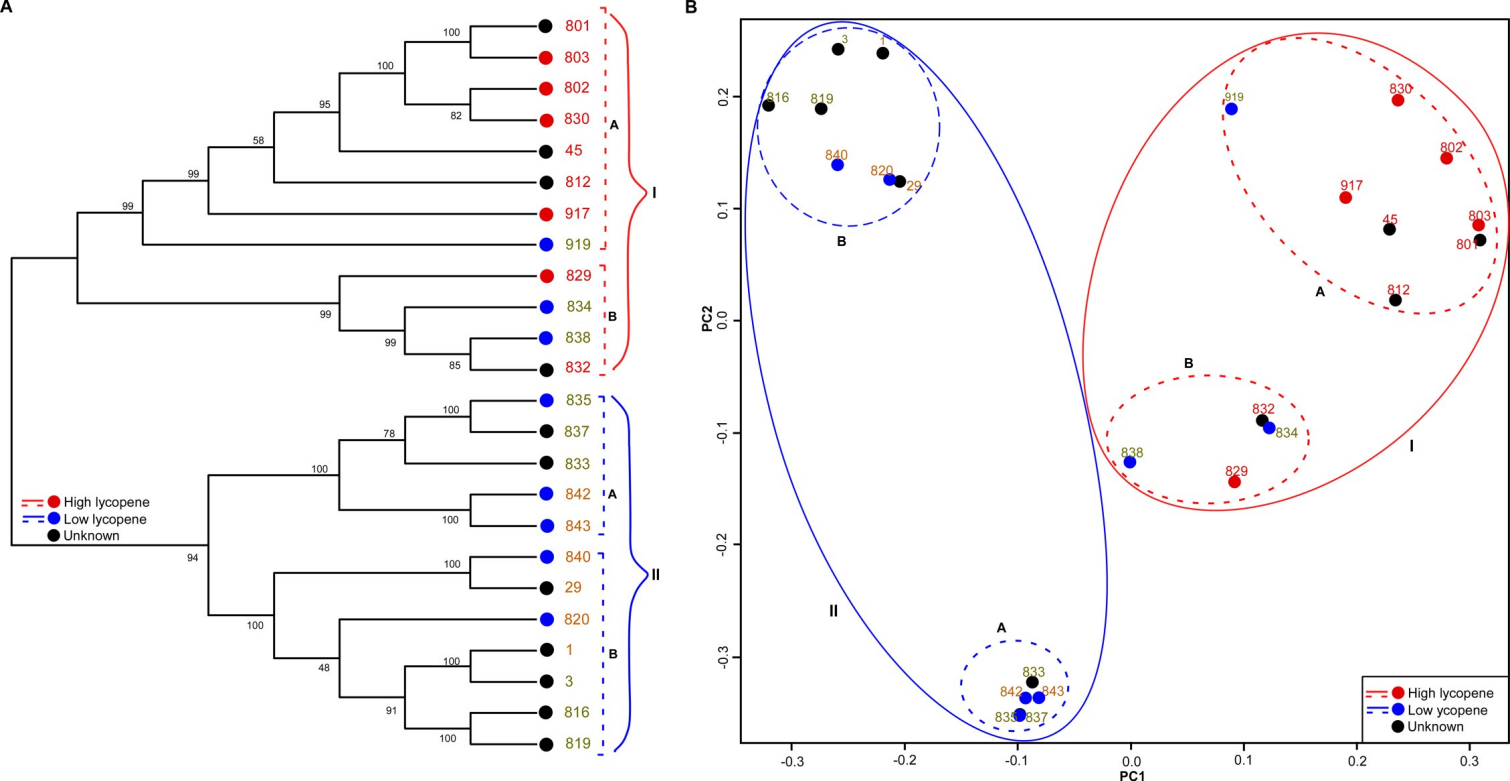
Phylogenetic tree using neighbor-joining (NJ) and principal coordinate analysis (PCA) of 24 watermelon inbred lines. (A) NJ dendrogram showing the genetic relationships among 24 watermelon lines based on 2369 SNP markers. Line names in red, olive, and orange represent the red, yellow, and orange flesh colors, respectively. Red or blue circles represent the estimated high or low lycopene levels, respectively, as mentioned in the Materials and Methods, while black circled lines indicate unknown lycopene levels. A total of 24 lines fall into the group I and group II major clusters, each comprising two other sub-clusters (A and B) as indicated. (B) PCA was performed with 2369 SNPs markers. The watermelon lines with high or low lycopene content fell into two groups, indicated as group I and II, respectively. The color and shape scheme is the same as those for the NJ analysis.

To determine the genetic relationships between the 24 different flesh-colored lines, a NJ tree was constructed using 2369 selected SNPs (S4 Table). According to the NJ tree (Fig 4A), the 24 lines could mainly be divided into two clusters: Cluster-I, consisting of all nine red flesh lines, as well as 3 yellow flesh lines (line 919, 834 and 838); and Cluster-II, consisting of 12 inbred lines with both yellow and orange flesh lines. Cluster-I could be further classified into two sub-clusters, A and B. In sub-cluster A, seven red flesh lines and one yellow flesh line clustered together, and in sub-cluster B, two red and two yellow flesh lines clustered together. Similarly, Cluster-II could also be classified into sub-clusters A and B, with both containing both yellow and orange fleshed lines. Recently, the effects of population structure and genetic diversity of watermelon on both genotypic and phenotypic levels were investigated [14, 24]. Discrepancies among studies may be due to difference in SNP marker types and grouping methods. Nevertheless, the NJ tree broadly separated the red flesh inbred lines with a high lycopene content (>333 μg/g) from the non-red (yellow or orange) flesh inbred lines with a low lycopene content (<65 μg/g) as noted in the carotenoid levels (Fig 1).

In addition, we performed PCA and found that the grouping results were consistent with those of the NJ tree analysis (Fig 4B). The results revealed that 24 inbred lines could be classified into four groups (groups A, B, C, and D) based on PC1 and PC2. Based on the flesh color phenotypic characteristics of the watermelon lines, the PCA generally classified the 24 inbred lines into the red flesh type with high lycopene content (Group A and B) and the non-red flesh type (yellow or orange) with low lycopene content (Group C and D) (Fig 4B). Unlike other fruit-related traits, flesh color is regulated by several alleles (*Y*, *y*, *y*^o^, *C*, and *I*) [27, 28], suggesting the involvement of a large number of loci. Other fruit-related traits, such as shape [49], skin color [50], or stripe pattern [51], have been found to be controlled by a small number of loci, having one (*O*), two (*G*^*-1*^, *G*^*-2*^), or three (*Dgo*, *S*, and *D*) alleles, respectively. Thus, genetic relationships within watermelon inbred lines, as inferred from genome-wide SNPs (Fig 4), would not be supported when correlating with other fruit-related traits of the lines, as listed in Table 1. We also assessed the population structure of the 24 inbred lines (S3 Fig). With no obvious inflexion points of the LnP(D) score, the Delta K value showed a peak at K = 3. Therefore, the 24 inbred lines could be classified into three clusters (S3 Fig): cluster I contained mainly red flesh types with high lycopene content genotypes, distributed into two sub-clusters (a and b); and clusters II and III both contained non-red flesh types (yellow and orange) with low lycopene genotypes (S3 Fig). These results are almost corroborated with the result analysis of the NJ tree and PCA. Therefore, both the NJ tree and PCA in this study, using SNP variants investigated in 24 inbred lines, further suggested that lycopene is a representative target fruit trait that distinguishes the red flesh type from the non-red flesh type.

We also determined the genome-wide distribution frequency of SNPs in three typical inbred lines with red (803: Cluster-IA), yellow (835: Cluster-IIA), and orange (840: Cluster-IIB) colors, grouped separately in both the PCA and NJ tree (Fig 4). As shown in the Circos plot, the distribution frequency of SNPs in three inbred lines (Circles D–F) was relatively higher in the centromeric region of the chromosomes than in the paracentromeric region, where coding sequences are largely distributed. In addition, comparison of the distribution frequency of SNPs between these three lines revealed distinct distribution patterns on chromosomes. In particular, the frequency of SNP distribution varied between inbred lines on chromosomes 2 (~20 Mb), 4 (~10 Mb), 5 (~10 Mb), 6 (~26 Mb), and 9 (~20 Mb) (Fig 2D–2F). This suggests that genetic similarity might be low between the inbred lines, further supporting the NJ-tree analysis and PCA (Fig 4A and 4B).

### Development, validation, and functional significance of polymorphic SNP markers associated with high lycopene content

**Fig 5:**
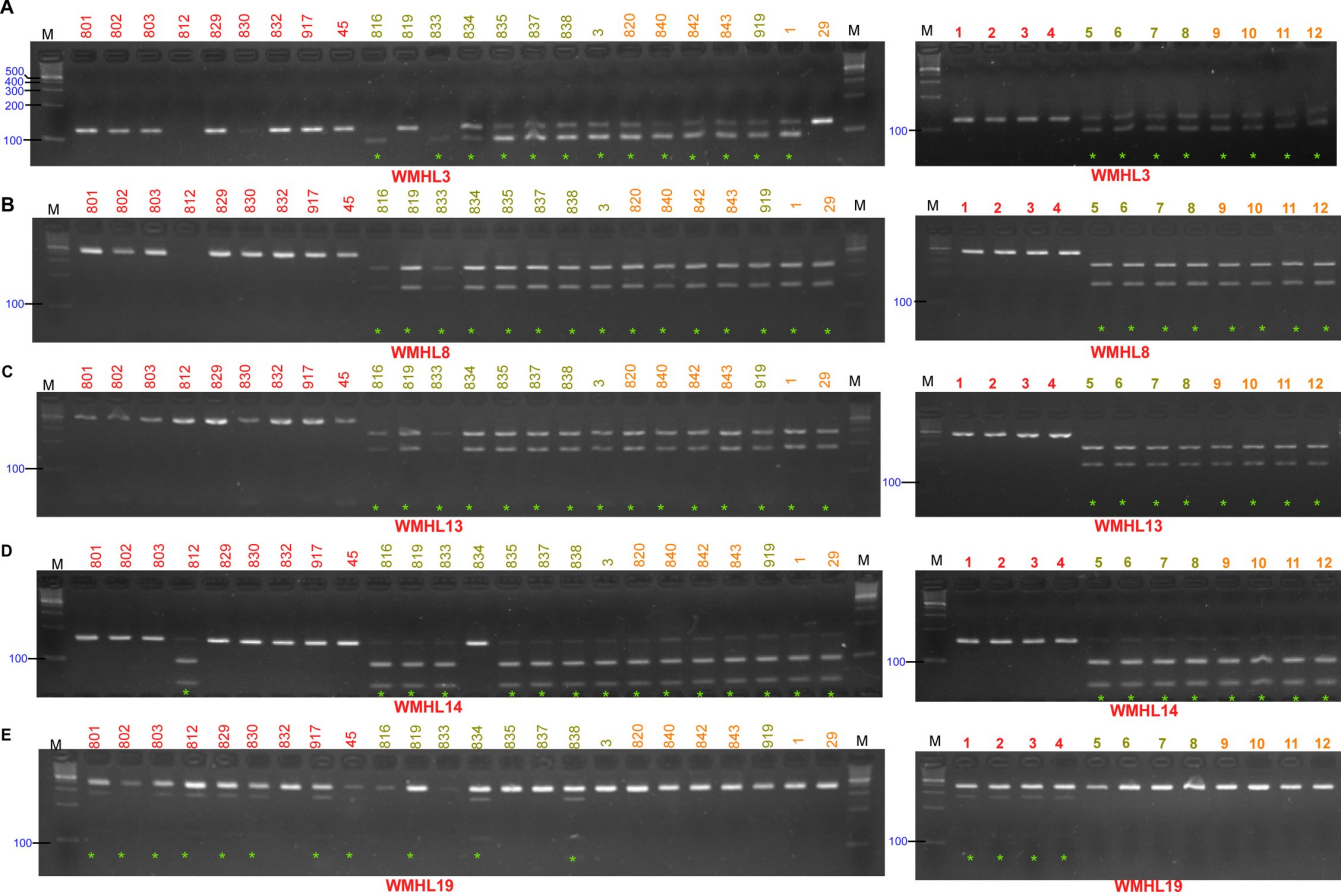
Gel pictures of four CAPS markers in 24 inbred lines and 12 commercial cultivars for the validation of five CAPS markers discovered during the genome resequencing. (A–E) Restriction digestion of PCR products of corresponding SNP markers are shown in gel pictures. M represents 100 bp markers and line names in red, olive, and orange represent the flesh colors of red, yellow, and orange, respectively (Table 1). On the right panel, the same CAPS markers were employed for the genotyping of 12 different commercial cultivars (S1 Table). The cleavage occurrences by selected restriction enzymes in the samples are indicated by a green asterisk.

The analysis of genetic diversity between 24 inbred lines revealed that the SNPs were able to generally distinguish between red flesh type with high lycopene content, and non-red flesh type, such as yellow or orange, with low lycopene cultivars. To validate the SNPs detected during resequencing (S4 Table), we randomly selected several SNPs on protein coding genes that presented polymorphism between red flesh and non-red flesh types. In total, 19 SNPs carrying 18 genes were selected, which were monomorphic among the red flesh type as well as among non-red flesh types. Simultaneously, they were converted to CAPS markers and primer sets were designed (Table 4). These 19 SNP markers were designated as WMHL1–19 (watermelon high lycopene). Using these 19 CAPS markers, PCR followed by restriction digestions were carried out on 24 inbred lines (Fig 5 and S4 Fig). Except for a few CAPS markers (WMHL2, WMHL15, and WMHL17) that generated no PCR product, the other 16 markers were consistent with the higher match rates > 0.92 (with an average of 0.99) between genotypes of SNPs and CAPS (Table 5).

**Table 4:** List of SNPs selected and converted into CAPS markers specific to lycopene-high (LH) and lycopene-low (LL) watermelons.

Marker name	SNP details					Descriptive information of the genes				Primer Sequence (5'–3')		Enzyme	Product size (bp)	
	Chr	Position (bp)	Ref. 97103	LH	LL	Accession	Name	Location	Amino acid change	Forward (5' to 3')	Reverse (5' to 3')		LH	LH
WMHL 1	Chr2	24493443	G	G	A	Cla020048	*Myosin 1*	Intron	-	TTCCCACCATTCGTAGACCG	AGATGAGGTCTTGGAAATGGCT	*Hind-III*	94	27, 67
WMHL 2	Chr2	24518665	T	T	C	Cla020046	*F-box/LRR-repeat protein 20*	Exon	Gly/Gly	GAAACTTGAATCTCGCCGGC	TGACATCCTTTGACAAGCTCTGT	*Acc-I*	59, 89	148
WMHL 3	Chr2	24751497	A	A	G	Cla020024	*Pre-mRNA-splicing factor RBM22*	Exon	Gln/Arg	CCTTGTATGTGGGCGGTCTT	AGGTTACAAAAGCGCATGCC	*XhoI*	123	20, 98
WMHL 4	Chr2	24830110	T	T	C	Cla020015	*Signal recognition particle protein*	Intron	-	GGGCAACTTGCACTCTTTAGT	TCAGAGAATTTGAAGGCCCGA	*SacI*	81	50, 31
WMHL 5	Chr2	24843410	A	A	G	Cla020013	*Vacuolar protein sorting-associated protein 27*	Exon	Ser/Ser	CTTCCACCTTTCTTGCCAAA	CCTGTTGCTGTTGAACCTGA	*ApeKI*	155	54, 101
WMHL 6	Chr2	24877311	T	T	C	Cla020012	*Cyclic nucleotide gated channel 9*	Intron	-	GCTTTTGCACGTGGGTAAAT	GCTCTGAATCACGCCTTTTC	*XbaI*	203	72, 130
WMHL 7	Chr2	24886838	C	C	T	Cla020011	*Leucine aminopeptidase 1*	Intron	-	CTGCACCAAGAACTGCTGCT	TGAATGAAAATAGAAATGGACGTA	*SalI*	94, 154	249
WMHL 8	Chr4	8923655	A	A	G	Cla005012	*Kinesin-like protein*	Intron	-	AAATAGTTGGCGGTGATTGC	GGCCATGACGTGACATTAAA	*BsrI*	468	171, 296
WMHL 9	Chr4	9807020	C	C	T	Cla006835	*Unknown Protein*	Exon	Glu/Lys	CCGGATCCTTGCTAGAGTCA	GTGTAGGACATTCCCGATGC	*AvaI*	90	33,57
WMHL 10	Chr4	9807385	A	A	G	Cla006835	*Unknown Protein*	Intron	-	CAACCCGCTCATGTAGTGAA	GGATGATCTTGGGCATGAGT	*SalI*	196	54, 141
WMHL 11	Chr4	10209760	T	T	C	Cla006847	*Unknown Protein (AHRD V1)*	Intron	-	ACCCCGTTGTGACTTAGTGT	ACAAAAGAAGAGGGTAAGCC	*BbsI*	45, 80	126
WMHL 12	Chr4	10826029	G	G	A	Cla006858	*Transcription initiation factor IIB-2*	Exon	Gly/Gly	CCGGAAACCTCACCATAAAG	CACGGTCAAGGGTATTGCTT	*BsrI*	53, 182	236
WMHL 13	Chr4	10838374	A	A	G	Cla006859	*Pentatricopeptide repeat-containing protein*	Exon	Leu/Pro	TAAATCCGGCCTCACACATT	GGGAAATGCAAACATATTGGA	*BsrI*	484	183, 301
WMHL 14	Chr5	20321987	A	A	G	Cla000884	*mRNA clone RTFL01-05-M08*	Intron	-	ATAAGGCGCATCGCTTAAAA	TGGTGTTCCCACCTTTTCTC	*EcoNI*	160	59, 101
WMHL 15	Chr5	21363473	A	A	G	Cla004209	*Trafficking protein particle complex subunit 3*	Intron	-	ACACACTCAAGAATGAACAGA	TGTGTGAGATTTCTTTTGGAGA	*Bsu36I*	146	35, 101
WMHL 16	Chr9	22158134	G	G	A	Cla012979	*Cleavage and polyadenylation specificity factor subunit 3*	Intron	-	ATGACCGTGGATGCACCTTT	CAGTGCTGCAATGCTCTTGG	*PstI*	134	26, 99
WMHL 17	Chr9	22239355	C	C	A	Cla012981	*Unknown Protein*	Exon	Ala/Ser	TCCCATTCCCATTACATGATCCC	TGAGAATCAGCCTGTGTCTGA	*PstI*	54, 95	149
WMHL 18	Chr10	26477503	T	C	T	Cla017825	*Zinc finger protein*	Exon	Val/Ala	TGCCACATTTTCGGGCTTTG	CGACGATCCATCTGACTGCA	*NruI*	50, 86	136
WMHL 19	Chr10	26681481	C	T	C	Cla017847	*Receptor-like kinase*	Intron	-	CAGTAGCCAACAGCAGGACA	TTCCTGGAGCGTCTTAAATCA	*NdeI*	119, 291	410

**Table 5:** Summary of result analysis of match rate between SNP and CAPS in 24 watermelon lines.

Marker name	Chromosome	Position	Ref	Lycopene-high lines									Lycopene-low lines															Match rate
				801	802	803	812	829	830	832	917	45	816	819	833	834	835	837	838	3	820	840	842	843	919	1	29	
WMHL1	Chr2	24493443	G	G/G	G/G	G/G	G/-^z^	G/G	G/G	G/G	G/G	G/G	A/A	G/G	A/A	R/R	A/A	A/A	A/A	A/A	A/A	A/A	A/A	A/A	A/A	A/A	G/G	1
WMHL2	Chr2	24518665	T	T/T	T/T	T/T	T/-	T/T	T/T	T/T	T/T	T/T	C/C	T/T	C/C	Y/C	C/T	C/C	C/C	C/C	C/C	C/C	C/C	C/C	C/C	C/C	T/T	0.92
WMHL3	Chr2	24751497	A	A/A	A/A	A/A	A/-	A/A	A/A	A/A	A/A	A/A	G/G	A/A	G/G	R/R	G/G	G/G	G/G	G/G	G/G	G/G	G/G	G/G	G/G	G/G	A/A	1
WMHL4	Chr2	24830110	T	T/T	T/T	T/T	T/-	T/T	T/T	T/T	T/T	T/T	T/T	T/T	C/C	Y/Y	C/C	C/C	C/C	C/C	C/C	C/C	C/C	C/C	C/C	C/C	T/T	1
WMHL5	Chr2	24843410	A	A/A	A/A	A/A	A/A	A/A	A/A	A/A	A/A	A/A	A/A	A/A	G/G	R/R	G/G	G/G	G/G	G/G	G/G	G/G	G/G	G/G	G/G	G/G	A/A	1
WMHL6	Chr2	24877311	T	T/T	T/T	T/T	T/T	T/T	T/T	T/T	T/T	T/T	T/T	T/T	C/C	Y/Y	C/C	C/C	C/C	C/C	C/C	C/C	C/C	C/C	C/C	C/C	T/T	1
WMHL7	Chr2	24886838	C	C/C	C/C	C/C	C/C	C/C	C/C	C/C	C/C	C/C	C/C	C/C	T/T	Y/Y	T/T	T/T	T/T	T/T	T/T	T/T	T/T	T/T	T/T	T/T	C/C	1
WMHL8	Chr4	8923655	A	A/A	A/A	A/A	A/-	A/A	A/A	A/A	A/A	A/A	G/G	G/G	G/G	G/G	G/G	G/G	G/G	G/G	G/G	G/G	G/G	G/G	G/G	G/G	G/G	1
WMHL9	Chr4	9807020	C	C/C	C/C	C/C	C/C	C/C	C/C	C/C	C/C	C/C	T/T	T/T	T/T	T/T	T/T	T/T	T/T	T/T	T/T	T/T	T/T	T/T	T/T	T/T	T/T	1
WMHL10	Chr4	9807385	A	A/A	A/A	A/A	A/A	A/A	A/A	A/A	A/A	A/A	G/G	G/G	G/G	G/G	G/G	G/G	G/G	G/G	G/G	G/G	G/G	G/G	G/G	G/G	G/G	1
WMHL11	Chr4	10209760	T	T/T	T/T	T/T	T/T	T/T	T/T	T/T	T/T	T/T	C/C	C/C	C/C	Y/C	C/C	C/C	C/C	C/C	C/C	C/C	C/C	C/C	C/C	C/C	C/C	0.96
WMHL12	Chr4	10826029	G	G/G	G/G	G/G	G/G	G/G	G/G	G/G	G/G	G/G	A/A	A/A	A/A	A/A	A/A	A/A	A/A	A/A	A/A	A/A	A/A	A/A	A/A	A/A	A/A	1
WMHL13	Chr4	10838374	A	A/A	A/A	A/A	A/A	A/A	A/A	A/A	A/A	A/A	G/G	G/G	G/G	G/G	G/G	G/G	G/G	G/G	G/G	G/G	G/G	G/G	G/G	G/G	G/G	1
WMHL14	Chr5	20321987	A	A/A	A/A	A/A	G/G	A/A	A/A	A/A	A/A	A/A	G/G	G/G	G/G	A/A	G/G	G/G	G/G	G/G	G/G	G/G	G/G	G/G	G/G	G/G	G/G	1
WMHL15	Chr5	21363473	A	A/A	A/A	A/A	G/-	A/A	A/A	A/A	A/A	A/A	G/G	G/G	G/G	A/A	G/G	G/G	G/G	G/G	G/G	G/-	G/G	G/G	G/G	G/G	G/G	1
WMHL16	Chr9	22158134	G	G/G	G/G	G/G	A/-	G/G	G/G	A/A	G/G	G/G	G/G	G/G	A/A	G/G	A/A	A/A	A/A	A/A	A/A	A/A	A/A	A/A	A/A	G/G	G/G	1
WMHL17	Chr9	22239355	C	C/C	C/C	C/C	A/-	C/C	C/C	C/C	C/C	C/C	C/C	C/C	A/A	C/C	A/A	A/A	A/A	A/A	A/A	A/A	A/A	A/A	A/A	**C/A**	C/C	0.96
WMHL18	Chr10	26477503	T	C/C	C/C	C/C	C/C	C/C	C/C	T/T	C/C	C/C	T/T	T/T	T/T	C/C	T/T	T/T	C/C	T/T	T/T	T/T	T/T	T/T	T/T	T/T	T/T	1
WMHL19	Chr10	26681481	C	T/T	T/T	T/T	T/T	T/T	T/T	C/C	T/T	T/T	C/C	C/C	C/C	T/T	C/C	C/C	T/T	C/C	C/C	C/C	C/C	C/C	C/C	C/C	C/C	1

Note:

^z^ the genotype was not available due to a failed PCR.

We also noted some discrepancies between SNP information (WMHL11 and WMHL17), which can be explained by a failure of restriction digestion or technical errors during resequencing, as observed in a previous study on watermelons [24]. With the high match rate (92%), the developed red flesh-type SNP markers in this study may be useful for the identification of high- and low-lycopene watermelon cultivars. To further check the reliability and applicability of these validated red flesh type or high-lycopene type specific SNPs, genotyping was performed on 12 different commercial cultivars comprising red (4), yellow (4), and orange (4) flesh color (S1 Table). The results revealed higher match rates (Fig 5, S4 Fig, and S5 Table), ranging from 0.83 to 1, between the genotypes of 19 developed CAPS markers and flesh color phenotypes in all tested inbred lines (S5 Table). Furthermore, the average match rate for all 19 CAPS markers was 0.97, indicating the high reliability and applicability of the high-lycopene type specific SNPs detected in this study.

To determine the functional significance of the 19 validated SNP-carrying genes, *in silico* expression profiling of these genes was performed using comparative watermelon transcript data (BioProject: PRJNA338036) from the same genes available in various fruit developmental stages of red-fleshed (LSW177) and pale-yellow-fleshed (COS) cultivated watermelons. The results revealed that these SNP-carrying genes presented preferential and stage-specific expression between LSW177 and COS. At 10 DPA, these genes were generally upregulated in pale yellow fleshed COS compared with LSW177. At the 26 DPA ripening stage, almost all genes displayed robust, uniform upregulated expression of their transcripts in both COS and LSW177, relative to other developmental stages. At the 18, 34, and 42 DPA stages, almost all SNP-carrying genes were significantly upregulated in the red flesh of LSW177 compared to that in COS (S5 Fig). Moreover, some of these SNP-carrying genes (WMHL 3, 9, 13, 17, and 18) contained non-synonymous substitutions in their exons (Table 4). These substitutions altered the amino acid residue of proteins, which could lead to the functional variation of those corresponding genes between red flesh and non-red flesh types. This suggests that these genes may be used as potential genic markers for the identification of cultivars with high lycopene contents. However, the results will need to be further validated using large segregating populations with more SNP markers associated with high lycopene content. The development of diagnostic, functional, or genic markers tightly linked to genes controlling target traits would broaden the successful application of MAS in watermelon breeding.

In watermelon, to date, several functional fruit trait-related markers have been identified; fruit shape (SUN-Cla011257), stripe pattern (wsb6-11), and flesh color (Clcyb.600 and Lcyb) [29, 30, 52]. However, the development of functional markers requires time for their subsequent identification, cloning, and interpretation, as in the case of Clcyb.600 and Lcyb [29, 30]. In this study, the basic principle for identifying and developing genic markers through WGRS was similar to that of resequencing methods in other studies [10, 24]. Our research showed that these strategies, which do not require laborious gene cloning and characterization, can be used to develop useful markers for MAS. It is also feasible to analyze large pieces of chromosomes, which can identify ample amounts of closely linked genic markers for MAS through WGRS. Therefore, the identification and development of molecular markers for watermelon breeding through WGRS is a simple and cost-effective approach.

### Analyses of high-density SNP regions specific to high lycopene content and red flesh color

Lycopene is a major carotenoid in red-fleshed watermelons. Lycopene has many positive effects on human physiology, including the prevention of cancer and oxidative damage. Therefore, lycopene research has been performed by various sectors, including the medical, nutrition, and cosmetics sectors. Liu et al. [33] mapped a single major QTL associated with the genetic regulation of flesh color and high lycopene content on chromosome 4 and three other minor QTLs for flesh color on chromosome 3, 6, and 11 of the watermelon genome. In addition, thus far, only two (Clcyb.600 and Lcyb) gene-specific markers have been developed for discriminating red flesh-type cultivars among canary yellow or orange lines [30]. In the present study, we investigated the genome-wide distribution of high-density SNP regions specific to high lycopene content and red flesh color and compared the results with the already reported markers flanking the QTL regions associated with flesh color and lycopene [8, 29, 30, 33]. A total of 2084 SNPs that can distinguish high-lycopene-content and red-fleshed cultivars among low-lycopene-content and non-red-fleshed cultivars were found at chromosomes 2, 3, 4, 5, 9, 11, and 0 (S6 Fig). On chromosome 4, there was a SNP hotspot that was concentrated at the 8.3 to 11.12 Mb region, comprising a sum of 1152 SNPs, where the major QTLs (flanked between WII04E08-38–WII04EBsaHI-6) for lycopene (LCYB4.1) and flesh color (FC4.1) as well as a CAPS marker of the *lycopene β-cyclase* (*LCYB*) gene (Cycl.600 and Lcyb) were identified [8, 29, 30, 33] (S6 Fig). In this SNP hotspot region, we noted the presence of more than 30 different genes, including the CDS of the *LCYB* gene (SNPs at physical locations of 8,886,348, 8,886,977, and 8,887,641 bp with PIC of 0.38), where all SNPs from these genes were found to be cosegregated with the flesh color and high-lycopene phenotypes of the inbred lines used in this study. As listed in S4 Table, during the genome identification of SNPs specific to high lycopene content, two genes were found nearest to *LCYB* at 8,721,803 (Cla005007: Unknown Protein) and 8,923,655 bp (Cla005012: Kinesin-like protein), with PICs of 0.37 and 0.38, respectively. The obtained SNP from *kinesin-like protein* (WMHL 8 at 8,923,655 bp) was confirmed to be cosegregated with the flesh color and high lycopene content upon validation (Table 4 and Fig 5), which suggests that linkage disequilibrium blocks might be present within individual genes on chromosomes among the selected populations in this study. This was similar to the findings of an earlier study, where Liu et al. [8] have reported that the genes within the QTL (FC4.1 and LCYB4.1) region in chromosome 4 could perfectly distinguish between red-fleshed and non-red-fleshed watermelons. In addition, it was also suggested that the QTLs (FC4.1 and LCYB4.1) in chromosome 4 might contain a single gene involved in the stimulation of the lycopene metabolic pathway; the lycopene content may be controlled by the very same gene or closely linked gene [8].

One SNP hotspot region was found in 22.1–23 Mb intervals on chromosome 3, consisting of a total of 43 SNPs. This SNP dense region was found to be closer to the already identified minor QTL (flanked between BVWS00048–WII03E09-92 at 27,914,506–25,064,560 bp, respectively) of flesh color (FC3.1) [33]. A QTL of red flesh color (flanked between RB1245–BNA23A) was previously mapped on chromosome 2 [32]. In the present study, we noted a high-density SNP region at 24–25 Mb intervals on chromosome 2. While validating some of the SNPs from chromosome 2 (WMHL 1–7), the results showed that they were cosegregated with the red flesh color phenotypes, suggesting that this region could be linked to red flesh color (Fig 5 and Table 5). Thus, the identified SNPs specific to high lycopene content and red flesh type in this study correspond to markers flanking the QTLs reported in earlier studies [8, 29, 30, 33]. Further, these results suggest that observed SNP hotspots may be associated with a chromosome block carrying the QTL region responsible for the high lycopene content and red flesh trait, and could be useful to explore the molecular markers for selecting that trait. By assessing the WGRS data of 24 inbred lines, the present study discovered a higher number of genic SNP markers at the genome-wide level (Table 3 and Fig 3). This could be an important resource for the watermelon research community to improve the genetic map construction and mining of more functional markers for higher lycopene content.

## Conclusions

In the present study, genome resequencing revealed significant genetic variations among different flesh-colored inbred lines in watermelons. By utilizing resequencing data, we identified numerous SNPs associated with flesh-color characteristics among all the inbred lines, which will be useful for the rapid development of diagnostic markers for MAS and fine mapping. Analysis of genetic diversity using 2369 SNPs mined on a genome-wide scale demonstrated practical significance, whereby two groups were found in the 24 inbred lines. Upon validation, the selected SNP/CAPS were able to distinguish between inbred lines with high (red) and low (yellow/orange) lycopene contents, and the results were correlated with the flesh color phenotype of each individual. Overall, for the first time, polymorphic SNPs were discovered in watermelons with high lycopene content. The SNPs identified from the current WGRS approach in watermelon may be utilized in population genetics, QTL mapping, and genomics in order to assist in the breeding of watermelons with high lycopene content.

## Supporting information

S1 Fig Photographs of longitudinal cross-sections of representative individuals for the flesh color categories of 24 inbred lines used in WGRS (A) and 12 commercial cultivars used in CAPS validation (B).
(PDF)

S2 Fig Functional annotation of genic SNPs by identification of corresponding transcripts and GO annotation. Identified transcripts are annotated into functional categories of cellular component, molecular function and biological process as indicated in the figure.
(PDF)

S3 Fig STRUCTURE analysis of 24 watermelon inbred lines based on 2369 SNP markers. (**A**) Determination of the optimal K-value (K = 1–8 with the admixture and correlated allele frequency models) based on five independent runs with burn-in period of 10,000 iterations followed by 10,000 Monte Carlo Markov Chain (MCMC) iterations according to a previous study [14]. The rate of change in the natural logarithm probability (LnP[D]) and its derived statistics ΔK for each K value are shown in left and right panel, respectively. (**B**) Population structure for the 24 watermelon inbred lines when K = 3. Each individual inbred line is indicated by a narrow vertical bar, which is partitioned into red, blue and green zones in proportion to the association coefficiencies to the 3 populations, such as the high lycopene content of cluster I comprising two other sub-clusters (A and B) and the low lycopene content of cluster II and III populations. The color and shape schemes are the same as those for the neighbor-joining (NJ) analysis in Fig 4A.
(TIF)

S4 Fig Agarose gel photos of validated CAPS markers discovered during genome resequencing. The name of validated CAPS markers (WMHL) are shown below their corresponding gel pictures. Numbers in lanes represents the DNA sample names of the inbred lines (Table 1) and commercial cultivars (S1 Table). M represents a 100 bp marker. Enzyme-cleaved DNA samples were indicated by “*green asterisk*”.
(PDF)

S5 Fig *In silico* expression profiles of 19 SNPs-carrying genes at developmental stages (10–42 DPA) of red-fleshed (LSW177) and pale-yellow-fleshed cultivated watermelon (COS) from the Cucurbit Expression Atlas (http://cucurbitgenomics.org/rnaseq). DPA and watermelon lines used for *in silico* expression profiling are indicated at the top and bottom of the heat map, respectively. A combined table showing the gene ID, marker name and gene description. The color scale at the top indicate up (*red*) or down (*green*) regulated expression.
(TIF)

S6 Fig Identification of high lycopene specific and red flesh color related SNP hotspots from genome resequencing data. Upper panel shows the position of SNP hotspots of different chromosomes. Lower panel describes the schematic representation of genetic linkage map for markers on chromosome 4 (WII04E08-38 to WII04EBsaHI-6) and Chromosome 3 (BVWS00048 to WII03E09-92) linked to flesh color (FC4.1 and FC3.1) or lycopene content (LCY4.1) according to previous studies [8, 33]. A CAPS marker position of *LCYB* gene (Cycl.600 and Lcyb) by Bang et al. [29, 30] marked by green line on chr4. Approximate physical locations of SNP hotspots (dashed blue box) of Chr4 (red oval symbol) and Chr3 (green circle symbol) are according to the map locus for high lycopene content and/or flesh color.
(TIF)

S1 Table List of 12 commercial watermelon cultivars (inbred lines) used in this study for CAPS marker validation and their representative fruit characteristics. (DOCX)

S2 Table Summary of the resequencing raw data results for 24 watermelon lines. (DOCX)

S3 Table Statistics of the sequencing trimmed data for 24 watermelon lines. (DOCX)

S4 Table Identified list of genome-wide SNPs between inbred lines with different flesh colors. (XLSX)

S5 Table The summary of result analysis of match rate between validated CAPS markers and SNPs in 12 watermelon commercial cultivars. (XLSX)
