# Supplementary figures and images for: Whole genome resequencing of watermelons to identify single nucleotide polymorphisms related to flesh color and lycopene content
Source: PLoS One. 2019 Oct 9;14(10):e0223441. doi: 10.1371/journal.pone.0223441 (PMC6785133; doi:10.1371/journal.pone.0223441)

A

Red

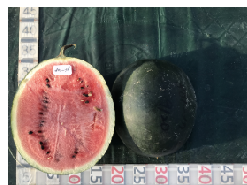

801

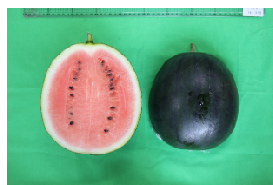

802

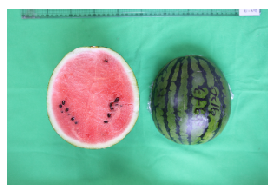

803

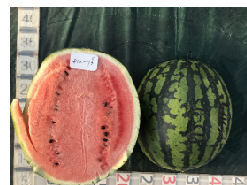

812

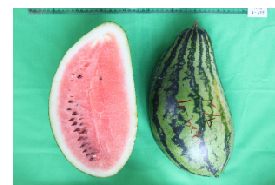

829

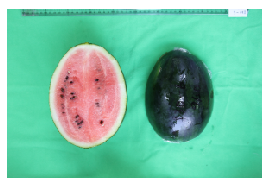

830

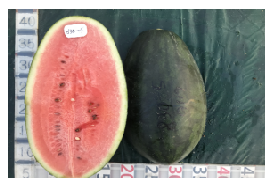

832

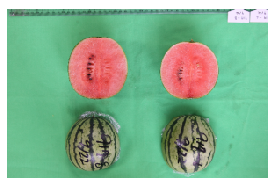

917

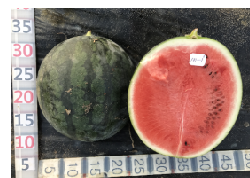

45

Yellow

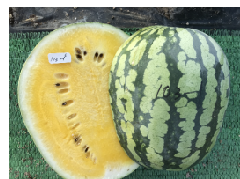

3

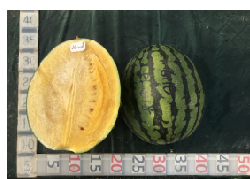

816

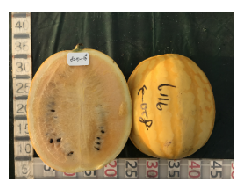

819

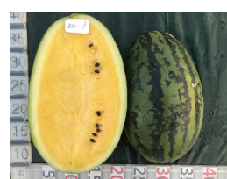

833

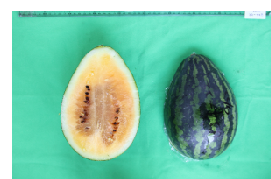

834

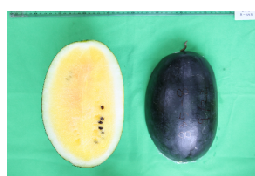

835

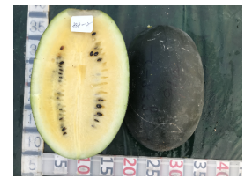

837

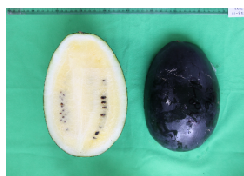

838

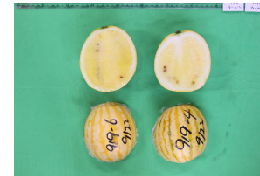

919

Orange

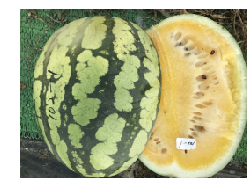

1

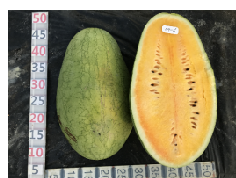

29

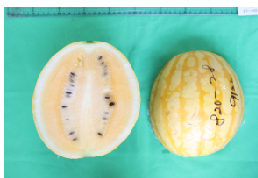

820

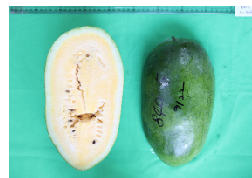

840

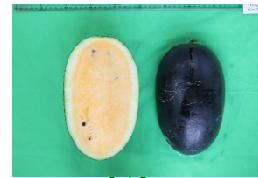

842

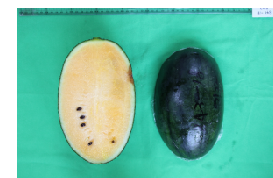

843

B

Red

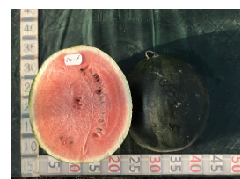

1

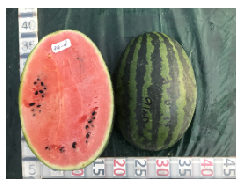

2

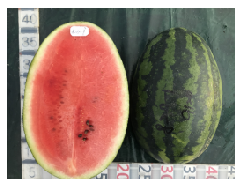

3

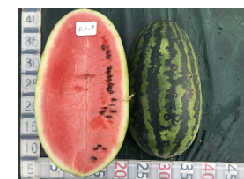

4

Yellow

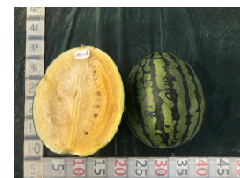

5

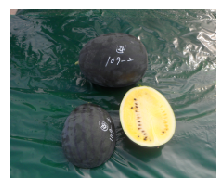

6

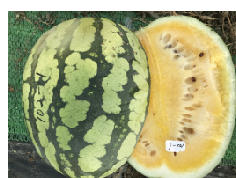

7

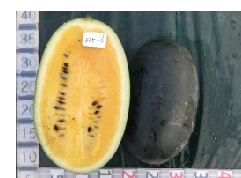

8

Orange

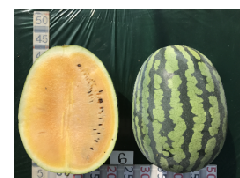

9

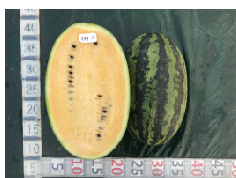

10

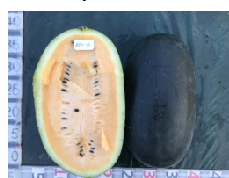

11

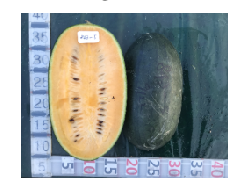

12

Supplement: S1 Fig — Photographs of longitudinal cross-sections of representative individuals for the flesh color categories of 24 inbred lines used in WGRS (A) and 12 commercial cultivars used in CAPS validation (B). (PDF) [file pone.0223441.s001.pdf]

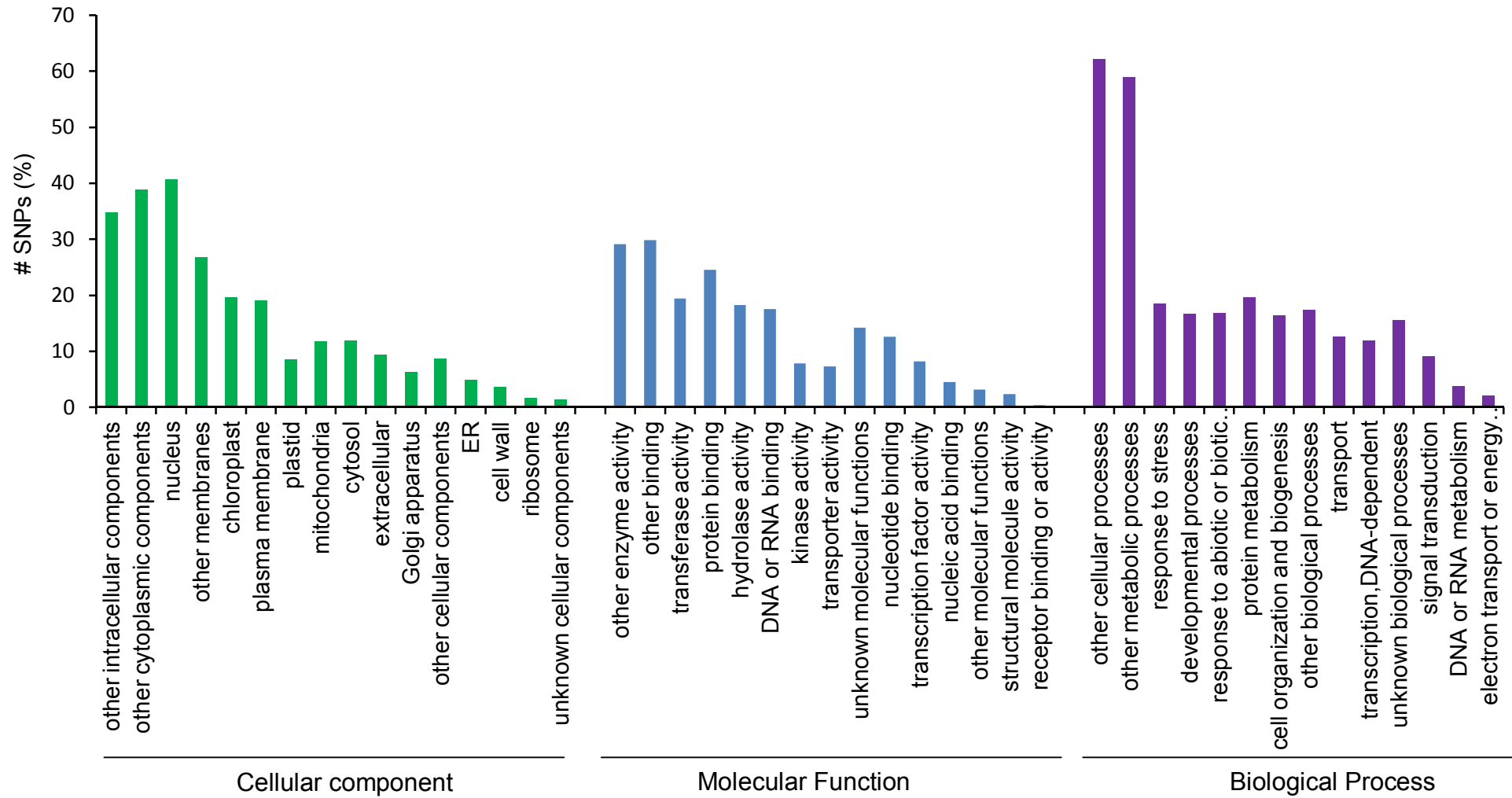

Supplement: S2 Fig — Identified transcripts are annotated into functional categories of cellular component, molecular function and biological process as indicated in the figure. (PDF) [file pone.0223441.s002.pdf]

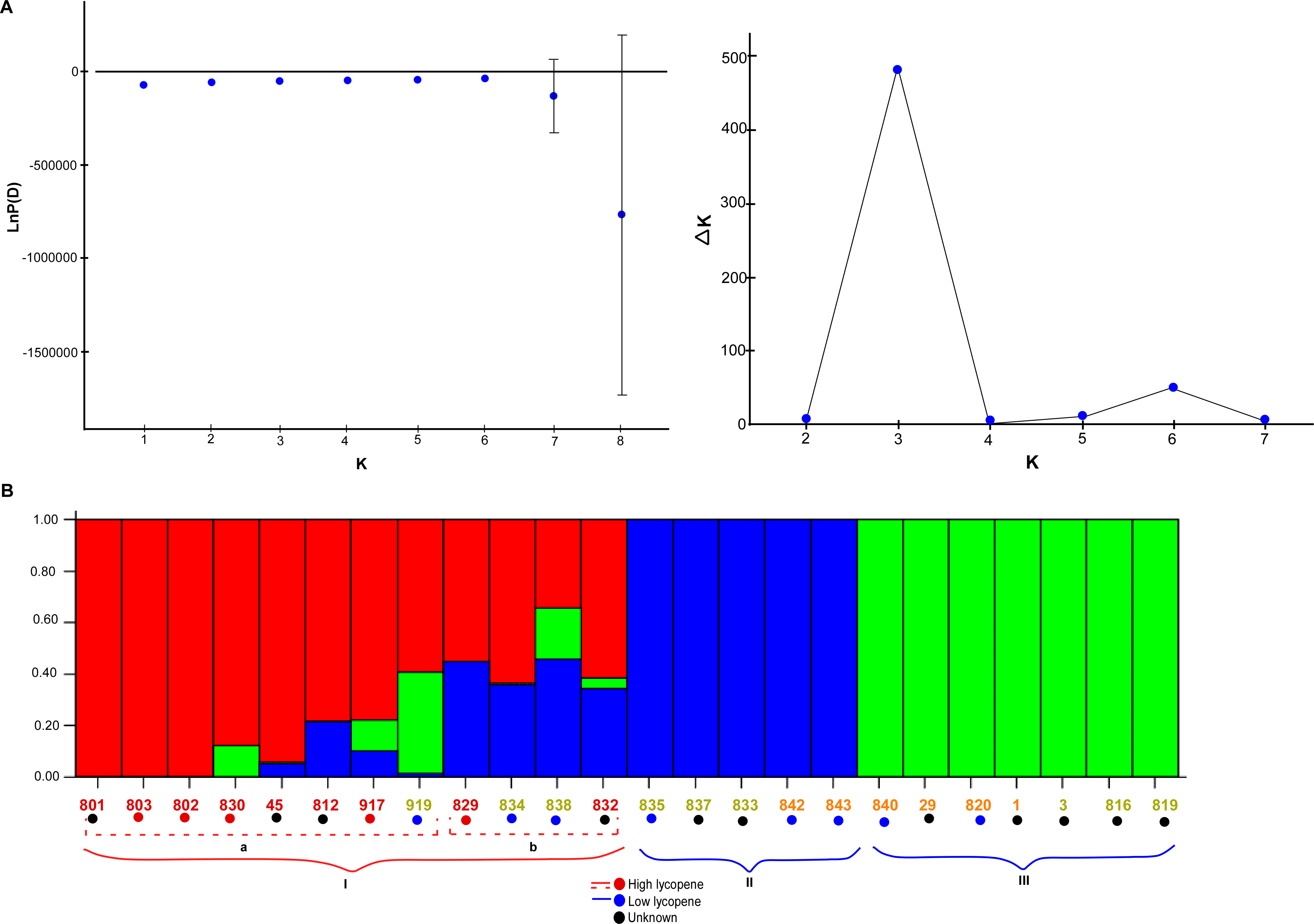

Supplement: S3 Fig — (A) Determination of the optimal K-value (K = 1–8 with the admixture and correlated allele frequency models) based on five independent runs with burn-in period of 10,000 iterations followed by 10,000 Monte Carlo Markov Chain (MCMC) iterations according to a previous study [14]. The rate of change in the natural logarithm probability (LnP[D]) and its derived statistics ΔK for each K value are shown in left and right panel, respectively. (B) Population structure for the 24 watermelon inbred lines when K = 3. Each individual inbred line is indicated by a narrow vertical bar, which is partitioned into red, blue and green zones in proportion to the association coefficiencies to the 3 populations, such as the high lycopene content of cluster I comprising two other sub-clusters (A and B) and the low lycopene content of cluster II and III populations. The color and shape schemes are the same as those for the neighbor-joining (NJ) analysis in Fig 4A. (TIF) [file pone.0223441.s003.tif]

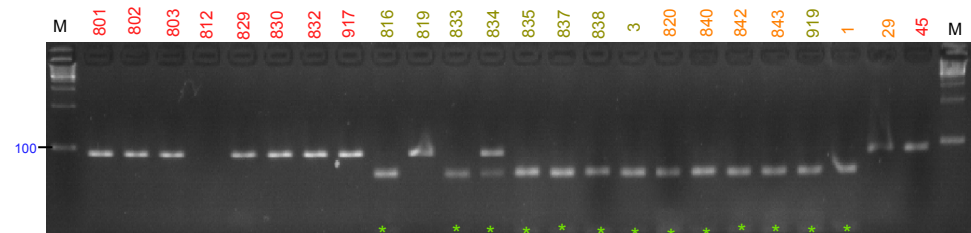

WMHL1

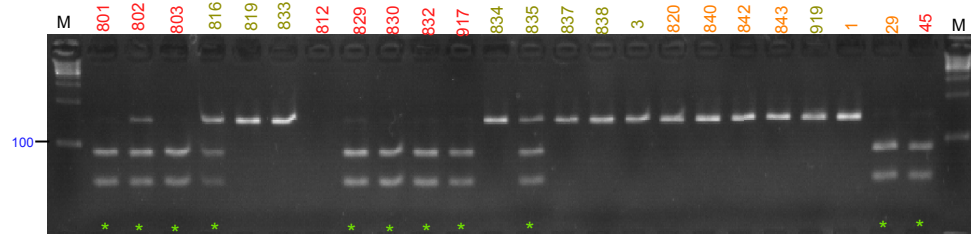

WMHL2

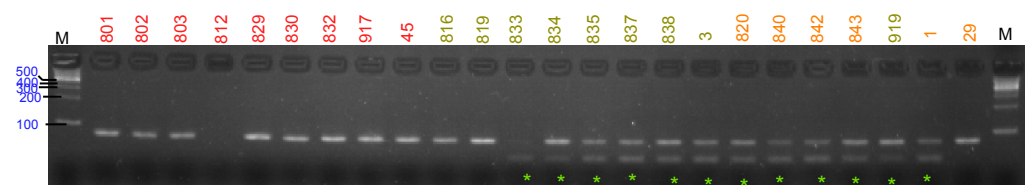

WMHL4

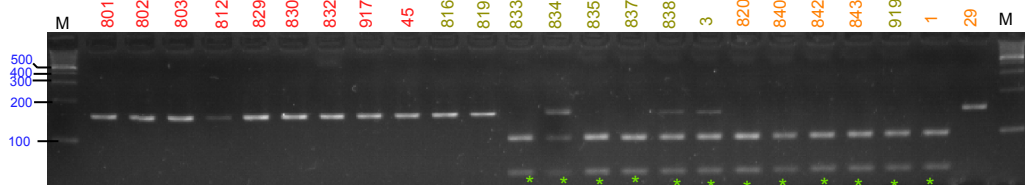

WMHL5

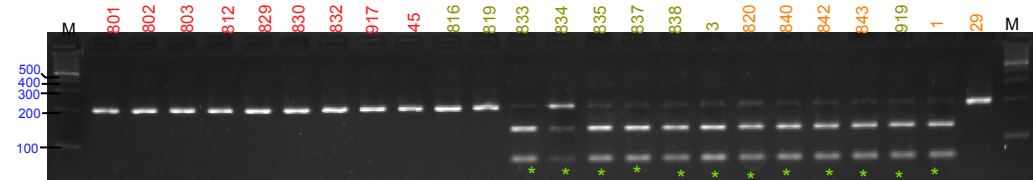

WMHL6

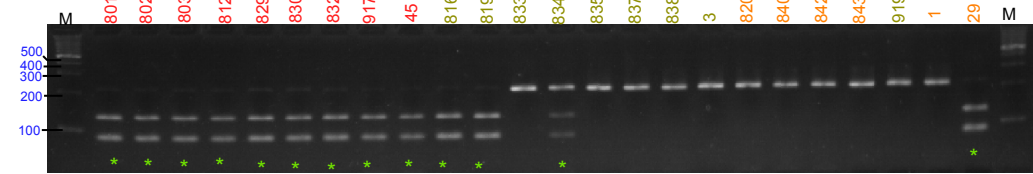

WMHL7

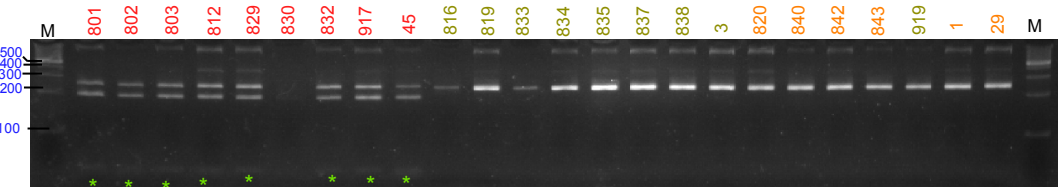

WMHL9

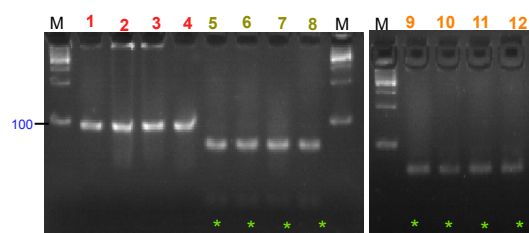

WMHL1

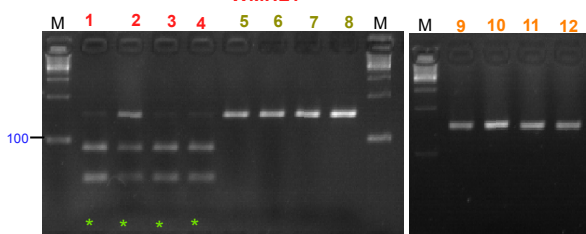

WMHL2

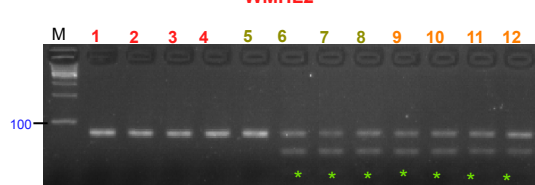

WMHL4

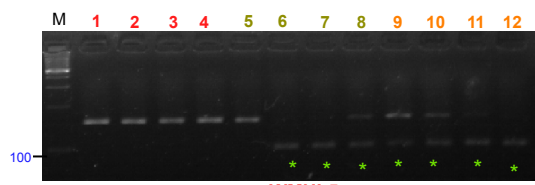

WMHL5

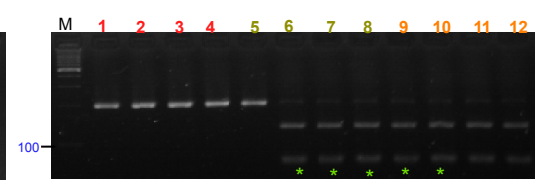

WMHL6

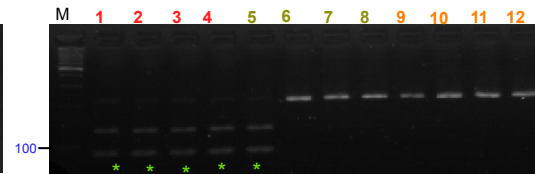

WMHL7

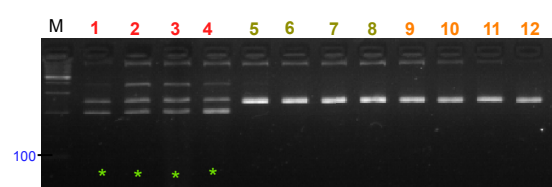

WMHL9

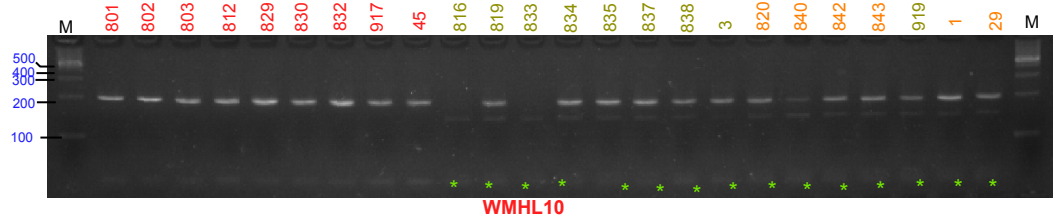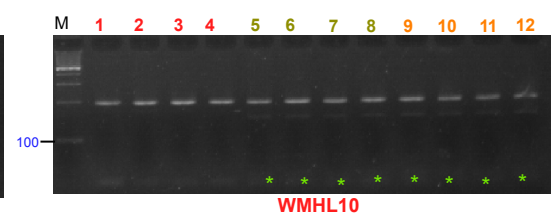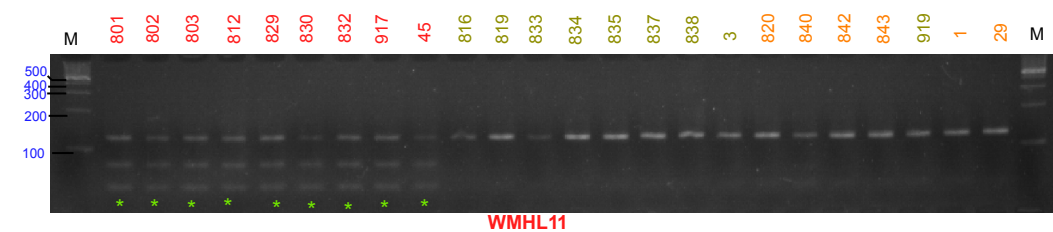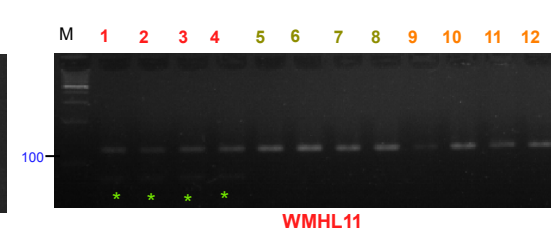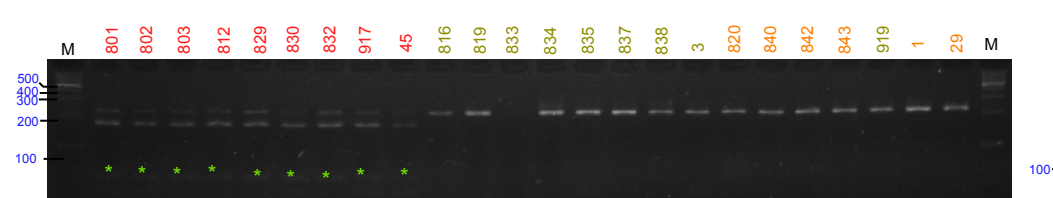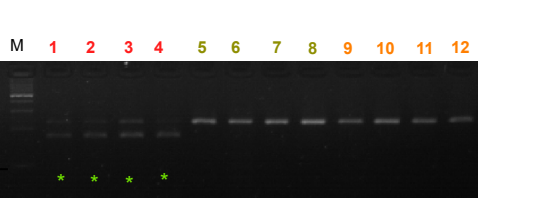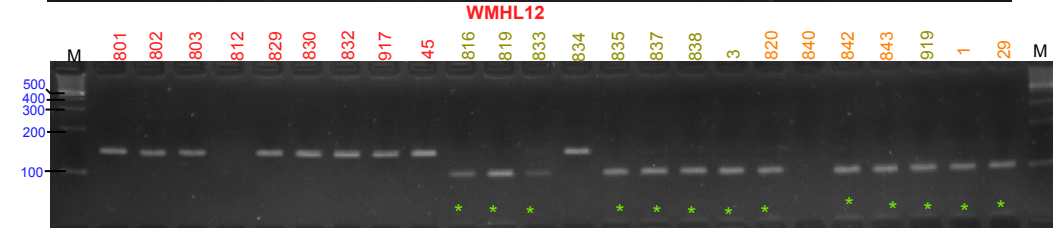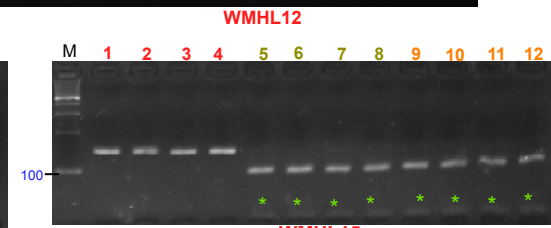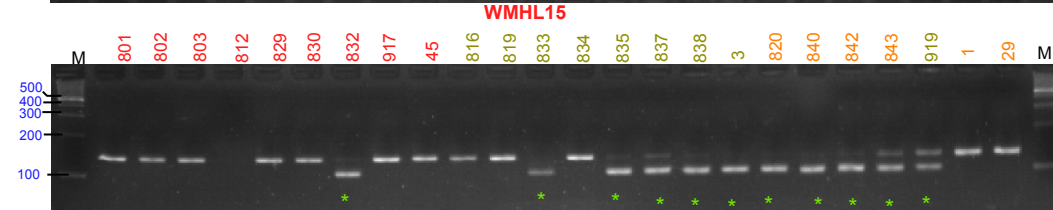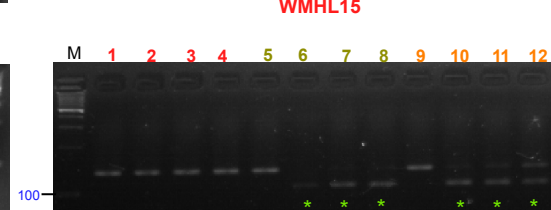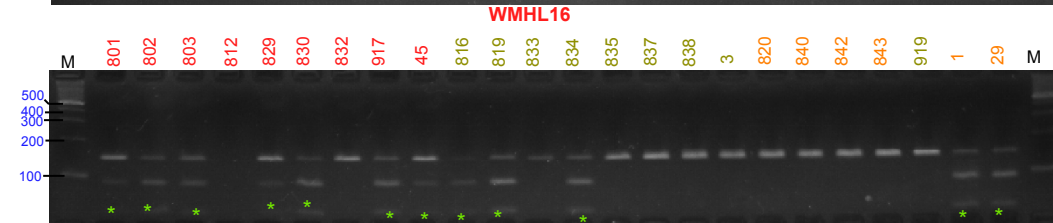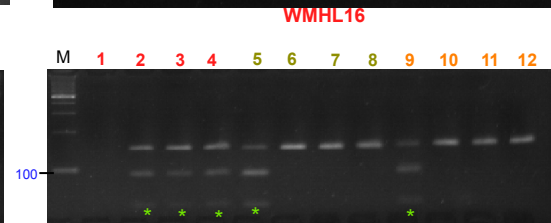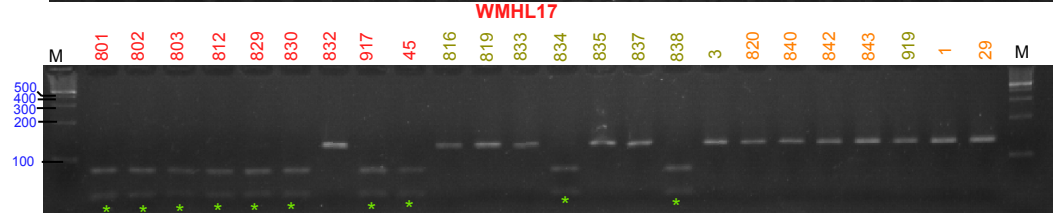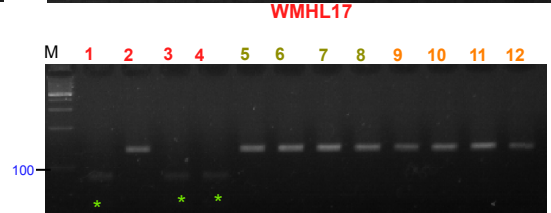

Supplement: S4 Fig — The name of validated CAPS markers (WMHL) are shown below their corresponding gel pictures. Numbers in lanes represents the DNA sample names of the inbred lines (Table 1) and commercial cultivars (S1 Table). M represents a 100 bp marker. Enzyme-cleaved DNA samples were indicated by “green asterisk”. (PDF) [file pone.0223441.s004.pdf]

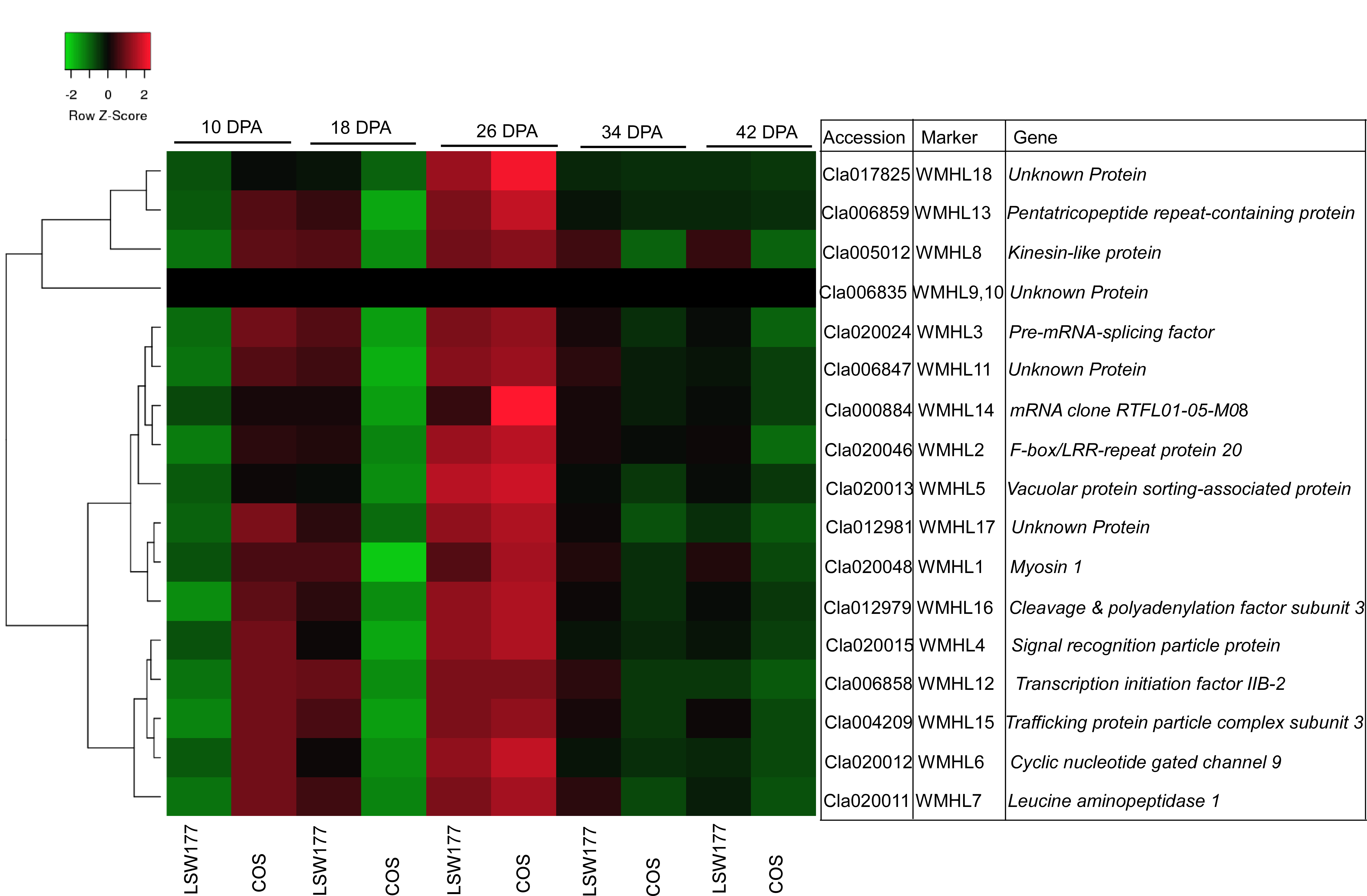

Supplement: S5 Fig — DPA and watermelon lines used for in silico expression profiling are indicated at the top and bottom of the heat map, respectively. A combined table showing the gene ID, marker name and gene description. The color scale at the top indicate up (red) or down (green) regulated expression. (TIF) [file pone.0223441.s005.tif]

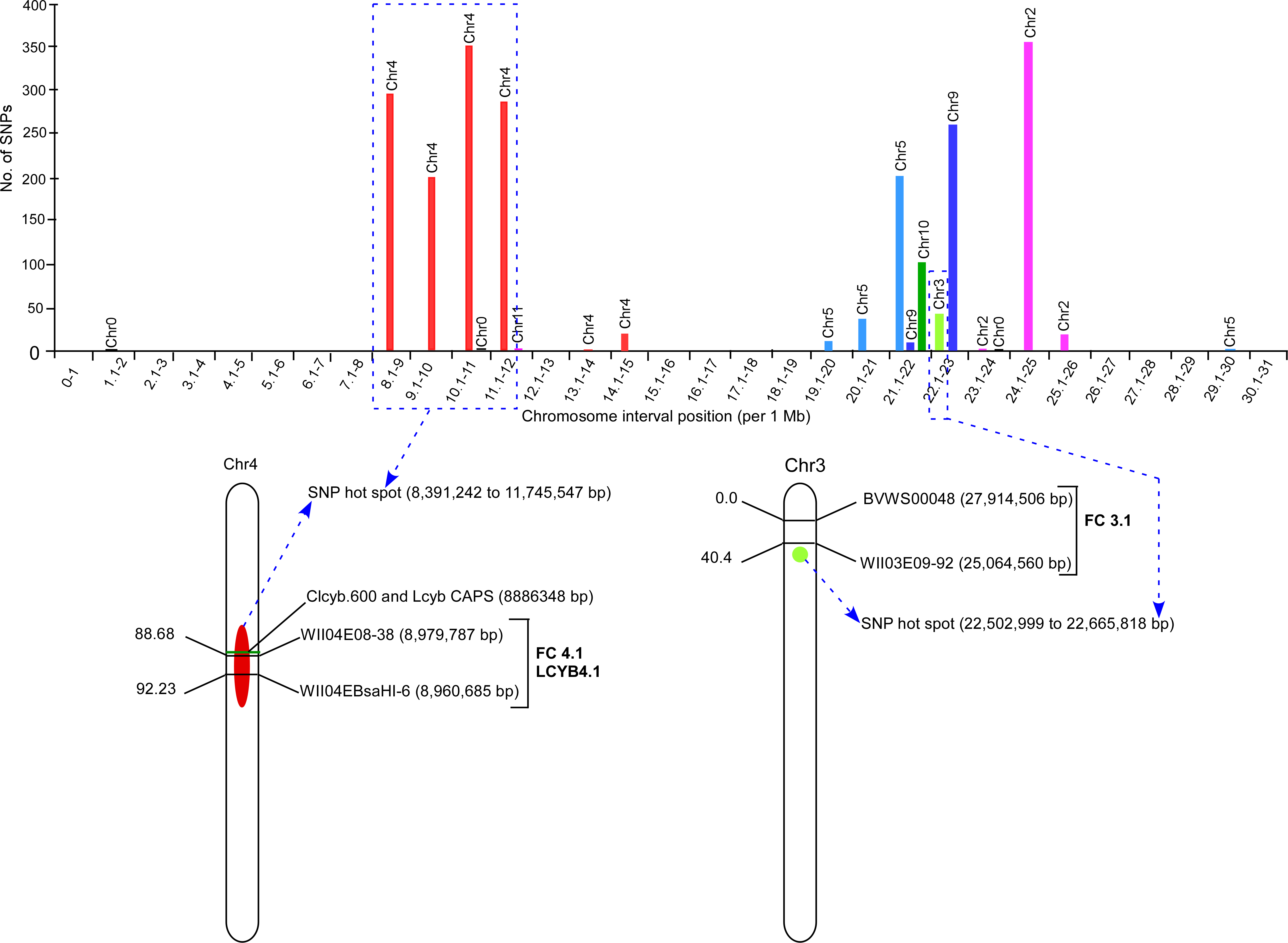

Supplement: S6 Fig — Upper panel shows the position of SNP hotspots of different chromosomes. Lower panel describes the schematic representation of genetic linkage map for markers on chromosome 4 (WII04E08-38 to WII04EBsaHI-6) and Chromosome 3 (BVWS00048 to WII03E09-92) linked to flesh color (FC4.1 and FC3.1) or lycopene content (LCY4.1) according to previous studies [8, 33]. A CAPS marker position of LCYB gene (Cycl.600 and Lcyb) by Bang et al. [29, 30] marked by green line on chr4. Approximate physical locations of SNP hotspots (dashed blue box) of Chr4 (red oval symbol) and Chr3 (green circle symbol) are according to the map locus for high lycopene content and/or flesh color. (TIF) [file pone.0223441.s006.tif]
