# Supplementary material for: Whole genome resequencing of watermelons to identify single nucleotide polymorphisms related to flesh color and lycopene content
Source: PLoS One. 2019 Oct 9;14(10):e0223441. doi: 10.1371/journal.pone.0223441 (PMC6785133; doi:10.1371/journal.pone.0223441)
Supplement: S1 Table — (DOCX) [file pone.0223441.s007.docx]

| **S. No** | **Cultivar name** | **Nursery** | **Fruit shape** | **Skin color** | **Stripe type** | **Flesh color** |
| --- | --- | --- | --- | --- | --- | --- |
| 1 | 1 | Hyundai Seeds | Circular | Black | Non (Solid) | Red |
| 2 | 2 | Hyundai Seeds | Elliptic | Green | Jubile | Red |
| 3 | 3 | Hyundai Seeds | Elliptic | Green | Jubile | Red |
| 4 | 4 | Hyundai Seeds | Elliptic | Green | Jubile | Red |
| 5 | 5 | Hyundai Seeds | Circular | Green | Jubile | Yellow |
| 6 | 6 | Hyundai Seeds | Circular | Black | Non (Solid) | Yellow |
| 7 | 7 | Hyundai Seeds | Circular | Green | Jubile | Yellow |
| 8 | 8 | Hyundai Seeds | Elliptic | Black | Non (Solid) | Yellow |
| 9 | 9 | Asia Seeds | Circular | Green | Jubile | Orange |
| 10 | 10 | Asia Seeds | Elliptic | Green | Jubile | Orange |
| 11 | 11 | Hyundai Seeds | Elliptic | Black | Non (Solid) | Orange |
| 12 | 12 | Hyundai Seeds | Elliptic | Black | Non (Solid) | Orange |

**Table S1**. List of 12 watermelon commercial cultivars (inbred lines) used in this study for CAPS marker validation and their representative fruit characteristics.
