# Supplementary material for: Whole genome resequencing of watermelons to identify single nucleotide polymorphisms related to flesh color and lycopene content
Source: PLoS One. 2019 Oct 9;14(10):e0223441. doi: 10.1371/journal.pone.0223441 (PMC6785133; doi:10.1371/journal.pone.0223441)
Supplement: S2 Table — (DOCX) [file pone.0223441.s008.docx]

**Table S2**. Summary of the re-sequencing raw data results for the 24 watermelon cultivars.

| **Samples** | **Total No. of Reads** | **Average length** | **Total length of the reads (bp)** | **Genome coverage** |
| --- | --- | --- | --- | --- |
| 1 | 39,711,334 | 101 | 4,010,844,734 | ≒18.87× |
|  | 39,711,334 | 101 | 4,010,844,734 |  |
| 3 | 37,117,136 | 101 | 3,748,830,736 | ≒17.64× |
|  | 37,117,136 | 101 | 3,748,830,736 |  |
| 29 | 38,982,438 | 101 | 3,937,226,238 | ≒18.53× |
|  | 38,982,438 | 101 | 3,937,226,238 |  |
| 43 | 39,316,526 | 101 | 3,970,969,126 | ≒18.69× |
|  | 39,316,526 | 101 | 3,970,969,126 |  |
| 45 | 38,836,542 | 101 | 3,922,490,742 | ≒18.46× |
|  | 38,836,542 | 101 | 3,922,490,742 |  |
| 50 | 34,948,664 | 101 | 3,529,815,064 | ≒16.61× |
|  | 34,948,664 | 101 | 3,529,815,064 |  |
| 54 | 35,737,606 | 101 | 3,609,498,206 | ≒16.99× |
|  | 35,737,606 | 101 | 3,609,498,206 |  |
| 57 | 36,684,641 | 101 | 3,705,148,741 | ≒17.44× |
|  | 36,684,641 | 101 | 3,705,148,741 |  |
| 514 | 40,885,779 | 101 | 4,129,463,679 | ≒19.43× |
|  | 40,885,779 | 101 | 4,129,463,679 |  |
| 579 | 36,430,573 | 101 | 3,679,487,873 | ≒17.32× |
|  | 36,430,573 | 101 | 3,679,487,873 |  |
| 802 | 40,505,746 | 101 | 4,091,080,346 | ≒19.25× |
|  | 40,505,746 | 101 | 4,091,080,346 |  |
| 803 | 37,484,112 | 101 | 3,785,895,312 | ≒17.82× |
|  | 37,484,112 | 101 | 3,785,895,312 |  |
| 819 | 37,719,811 | 101 | 3,809,700,911 | ≒17.93× |
|  | 37,719,811 | 101 | 3,809,700,911 |  |
| 820 | 41,319,897 | 101 | 4,173,309,597 | ≒19.64× |
|  | 41,319,897 | 101 | 4,173,309,597 |  |
| 829 | 40,049,810 | 101 | 4,045,030,810 | ≒19.04× |
|  | 40,049,810 | 101 | 4,045,030,810 |  |
| 830 | 40,010,393 | 101 | 4,041,049,693 | ≒19.02× |
|  | 40,010,393 | 101 | 4,041,049,693 |  |
| 834 | 34,839,599 | 101 | 3,518,799,499 | ≒16.56× |
|  | 34,839,599 | 101 | 3,518,799,499 |  |
| 835 | 39,858,295 | 101 | 4,025,687,795 | ≒18.94× |
|  | 39,858,295 | 101 | 4,025,687,795 |  |
| 838 | 36,069,187 | 101 | 3,642,987,887 | ≒17.14× |
|  | 36,069,187 | 101 | 3,642,987,887 |  |
| 840 | 39,553,769 | 101 | 3,994,930,669 | ≒18.80× |
|  | 39,553,769 | 101 | 3,994,930,669 |  |
| 842 | 41,950,305 | 101 | 4,236,980,805 | ≒19.94× |
|  | 41,950,305 | 101 | 4,236,980,805 |  |
| 843 | 39,790,654 | 101 | 4,018,856,054 | ≒18.91× |
|  | 39,790,654 | 101 | 4,018,856,054 |  |
| 917 | 39,683,075 | 101 | 4,007,990,575 | ≒18.86× |
|  | 39,683,075 | 101 | 4,007,990,575 |  |
| 918 | 38,984,154 | 101 | 3,937,399,554 | ≒18.53× |
|  | 38,984,154 | 101 | 3,937,399,554 |  |
