# Supplementary material for: Whole genome resequencing of watermelons to identify single nucleotide polymorphisms related to flesh color and lycopene content
Source: PLoS One. 2019 Oct 9;14(10):e0223441. doi: 10.1371/journal.pone.0223441 (PMC6785133; doi:10.1371/journal.pone.0223441)
Supplement: S3 Table — (DOCX) [file pone.0223441.s009.docx]

**Table S3**. Statistics of the sequencing trimmed data for the 24 watermelon cultivars.

| **Samples** | **Total No. of Reads (2 paired ends)** | **Average length** | **Total length of the reads (bp)** | **Trimmed/Raw read** | **Genome coverage** |
| --- | --- | --- | --- | --- | --- |
| 1 | 31,190,146 | 83.81 | 2,614,074,286 | 65.18% | ≒12.14× |
|  | 31,190,146 | 81.65 | 2,546,614,922 | 63.49% |  |
| 3 | 29,521,700 | 79.65 | 2,351,364,863 | 62.72% | ≒11.13× |
|  | 29,521,700 | 80.52 | 2,377,208,131 | 63.41% |  |
| 29 | 30,413,021 | 78.82 | 2,397,067,853 | 60.88% | ≒11.37× |
|  | 30,413,021 | 80.01 | 2,433,413,764 | 61.81% |  |
| 43 | 30,955,548 | 80.16 | 2,481,388,501 | 62.49% | ≒11.76× |
|  | 30,955,548 | 81.24 | 2,514,719,908 | 63.33% |  |
| 45 | 30,353,122 | 79.33 | 2,407,928,441 | 61.39% | ≒11.38× |
|  | 30,353,122 | 79.99 | 2,427,870,676 | 61.90% |  |
| 50 | 27,447,026 | 77.65 | 2,131,334,730 | 60.38% | ≒10.18× |
|  | 27,447,026 | 79.94 | 2,193,996,614 | 62.16% |  |
| 54 | 27,825,608 | 77.65 | 2,160,642,871 | 59.86% | ≒10.29× |
|  | 27,825,608 | 79.53 | 2,212,914,517 | 61.31% |  |
| 57 | 29,084,994 | 80.54 | 2,342,418,545 | 63.22% | ≒11.02× |
|  | 29,084,994 | 80.45 | 2,339,989,764 | 63.16% |  |
| 514 | 31,358,921 | 79.18 | 2,482,926,021 | 60.13% | ≒11.77× |
|  | 31,358,921 | 80.31 | 2,518,470,472 | 60.99% |  |
| 579 | 29,003,907 | 76.7 | 2,224,665,538 | 60.46% | ≒10.77× |
|  | 29,003,907 | 81.06 | 2,351,032,407 | 63.90% |  |
| 802 | 32,313,069 | 83.7 | 2,704,760,307 | 66.11% | ≒12.62× |
|  | 32,313,069 | 82.29 | 2,658,949,395 | 64.99% |  |
| 803 | 29,606,043 | 81.83 | 2,422,718,245 | 63.99% | ≒11.31× |
|  | 29,606,043 | 80.53 | 2,384,290,805 | 62.98% |  |
| 819 | 30,619,352 | 81.75 | 2,503,283,803 | 65.71% | ≒11.77× |
|  | 30,619,352 | 81.56 | 2,497,458,044 | 65.56% |  |
| 820 | 32,215,444 | 82.42 | 2,655,090,391 | 63.62% | ≒12.42× |
|  | 32,215,444 | 81.48 | 2,625,014,948 | 62.90% |  |
| 829 | 31,528,699 | 83.92 | 2,645,841,567 | 65.41% | ≒12.26× |
|  | 31,528,699 | 81.4 | 2,566,464,321 | 63.45% |  |
| 830 | 31,724,495 | 81.06 | 2,571,601,965 | 63.64% | ≒12.16× |
|  | 31,724,495 | 81.88 | 2,597,707,374 | 64.28% |  |
| 834 | 27,869,412 | 79.81 | 2,224,258,617 | 63.21% | ≒10.55× |
|  | 27,869,412 | 81.06 | 2,259,171,512 | 64.20% |  |
| 835 | 31,743,447 | 82.28 | 2,611,935,473 | 64.88% | ≒12.32× |
|  | 31,743,447 | 82.61 | 2,622,349,835 | 65.14% |  |
| 838 | 28,519,734 | 79.02 | 2,253,605,757 | 61.86% | ≒10.68× |
|  | 28,519,734 | 80.16 | 2,286,007,015 | 62.75% |  |
| 840 | 32,058,034 | 80.86 | 2,592,292,714 | 64.89% | ≒12.27× |
|  | 32,058,034 | 81.77 | 2,621,343,148 | 65.62% |  |
| 842 | 32,617,089 | 82.81 | 2,701,131,219 | 63.75% | ≒12.49× |
|  | 32,617,089 | 79.98 | 2,608,792,288 | 61.57% |  |
| 843 | 31,806,273 | 83.97 | 2,670,775,810 | 66.46% | ≒12.46× |
|  | 31,806,273 | 82.47 | 2,622,989,090 | 65.27% |  |
| 917 | 31,644,391 | 82.79 | 2,619,972,132 | 65.37% | ≒12.29× |
|  | 31,644,391 | 82.31 | 2,604,750,546 | 64.99% |  |
| 918 | 31,528,071 | 80.61 | 2,541,608,848 | 64.55% | ≒12.03× |
|  | 31,528,071 | 81.56 | 2,571,273,454 | 65.30% |  |
